# Semaphorin-7A on Exosomes: A Promigratory Signal in the Glioma Microenvironment

**DOI:** 10.3390/cancers11060758

**Published:** 2019-05-30

**Authors:** Ivana Manini, Maria Elisabetta Ruaro, Riccardo Sgarra, Anna Bartolini, Federica Caponnetto, Tamara Ius, Miran Skrap, Carla Di Loreto, Antonio Paolo Beltrami, Guidalberto Manfioletti, Daniela Cesselli

**Affiliations:** 1 Department of Medicine, University of Udine, Piazzale S. Maria della Misericordia 15, 33100 Udine, Italy; anna.bartolini90@hotmail.it (A.B.); federica.caponnetto25@gmail.com (F.C.); carla.diloreto@uniud.it (C.D.L.); antonio.beltrami@uniud.it (A.P.B.); daniela.cesselli@uniud.it (D.C.); 2 Department of Life Sciences, University of Trieste, Via Giorgieri 5, 34127 Trieste, Italy; rsgarra@units.it (R.S.); manfiole@units.it (G.M.); 3 Department of Neurosurgery, University Hospital of Udine, Piazzale S. Maria della Misericordia 15, 33100 Udine, Italy; tamara.ius@gmail.com (T.I.); skrap@aoud.sanita.fvg.it (M.S.)

**Keywords:** glioblastoma microenvironment, exosomes, Semaphorin 7A, integrin β1/FAK signalling, motility

## Abstract

Exosomes are one of the most important mediators of the cross talk occurring between glioma stem cells (GSCs) and the surrounding microenvironment. We have previously shown that exosomes released by patient-derived glioma-associated stem cells (GASC) are able to increase, in vitro, the aggressiveness of both GSC and glioblastoma cell lines. To understand which molecules are responsible for this tumour-supporting function, we performed a descriptive proteomic analysis of GASC-exosomes and identified, among the others, Semaphorin7A (SEMA7A). SEMA7A was described as a promigratory cue in physiological and pathological conditions, and we hypothesised that it could modulate GSC migratory properties. Here, we described that SEMA7A is exposed on GASC-exosomes’ surface and signals to GSC through Integrin β1. This interaction activates focal adhesion kinase into GSC and increases their motility, in our patient-based in vitro model. Our findings suggest SEMA7A-β1-integrin as a new target to disrupt the communication between GSCs and the supporting microenvironment.

## 1. Introduction

Despite a state-of-the art treatment, the median overall survival of glioblastoma multiforme (GBM)’s patients is 14 months [1]. The lack of therapeutic efficacy is due to the great intertumour and intratumour cellular and molecular heterogeneity, biological aggressiveness, the ability to develop drug resistance, as well as the infiltrative nature of tumour cells into the surrounding brain parenchyma [2]. 

Infiltration makes impossible a radical surgery thus favouring recurrences within 1 to 2 cm from the original tumour mass, appearing few months after the first diagnosis and treatment or being already present at the time of the initial presentation [3]. Moreover, single infiltrating cells are often spread throughout the entire brain parenchyma, escaping from surgery and treatment [4]. 

Several studies suggest that GBM’s tumour bulk contains a subpopulation of self-renewing and highly tumorigenic stem cells named glioma stem cells (GSCs) [5,6], with tumour-initiating properties that contribute to tumour growth and malignancy [7] through their sustained proliferation, invasion, stimulation of angiogenesis, ability to suppress immune responses and to develop resistance to therapy [8,9]. As normal neural stem cells, GSCs reside in specialised niches, where they receive maintenance stimuli and protection by interacting with the components of the surrounding milieu [5]. The tumour microenvironment contains various nontumoral cell types, such as endothelial cells, tumour-associated macrophages (TAMs), pericytes, resident astrocytes, as well as extracellular matrix components and secreted molecules such as growth and differentiation factors [5,6,7]. GSCs actively remodel the microenvironment, establishing a continuous cross-talk, which maintains and contributes to disease progression. Since the dialogue between GSCs and microenvironment gives a remarkable support to GBM, identification of key actors playing in this scenario has become increasingly challenging.

Growing evidence highlight that extracellular vesicles (EV) are one of the most important mediators of the cross-talk occurring in the tumour environment [10,11]. EV comprise a heterogeneous group of vesicles with sizes ranging from 100 to 1000 nm that play an important role in intercellular communication, by transporting complex cargoes [12,13]. Exosomes represent a subpopulation of EV (50–150 nm) generated into multivesicular endosomes that direct bioactive molecules (RNA, microRNA, proteins and lipids) to specific target cells, thereby altering their functions and/or reprogramming their genomic landscape [14]. 

In glioma, communication mediated by exosomes has not been extensively studied. However, some reports show the impact of tumour cell-derived exosomes on tumour biology [15,16]. 

Studies in breast and gastric cancer [17,18] have provided important information on the role of stromal-derived exosomes in enhancing growth, invasion and motility of tumour cells, although the molecular mechanisms by which exosomes exert their tumour-promoting role was not elucidated. 

We have previously established a protocol to isolate a population of stem cells, named glioma-associated stem cells (GASC) from human low- and high-grade gliomas. GASC are characterised by an undifferentiated mesenchymal phenotype, clonogenicity and multipotency, in vitro [19,20]. This cell population is devoid of tumour-initiating property in vivo, and it do not show genetic aberrations characterising the tumour of origin, nevertheless GASC show the ability to grow in an anchorage-independent way. For these reasons, GASC represent bona fide glioma stromal cells, residing in tumour microenvironment.

We have also demonstrated that GASC are characterised by the ability to support the biological aggressiveness of tumour cells (both GSCs and immortalised tumour cell lines) in vitro, including their motility, through the release of exosomes [19].

To understand which signals present in GASC exosomes are able to increase the aggressiveness of GSCs, we analysed the protein content of exosomes isolated from GASC-conditioned medium and found that, among other proteins, they transport Semaphorin 7A (SEMA7A). 

Semaphorins are a family of secreted and membrane-bound glycoproteins, known as important guidance signals in development of the central nervous system [21]. Semaphorins are expressed also outside the Central Nervous System CNS and are involved in many other biological processes, including regulation of cell survival, cell adhesion, motility, angiogenesis, immune response regulation and tumour progression [22,23].

SEMA7A is traditionally described as a guidance cue for axon and neurite outgrowth [24,25] and its biological effect could be mediated by interactions with two surface receptors: heterodimeric integrins α1β1 and Plexin C1 [26]. In human melanocytes, SEMA7A-integrin β1 interaction is described as a promoter of adhesion and dendricity [27], while the binding of SEMA7A to PlexinC1 inhibits dendrites formation. SEMA7A is also responsible for monocytes migration, during inflammatory processes [28] and is involved in the progression of ductal carcinoma, mediating growth, motility and invasion [29].

Here we demonstrate that SEMA7A carried by GASC-derived exosomes enhances the motility of GSCs, and suggest the mechanism of action via interaction with β1-integrin, expressed on GSCs surface.

## 2. Results

### 2.1. Characterisation of GASC-Derived Exosomes

**Figure 1:**
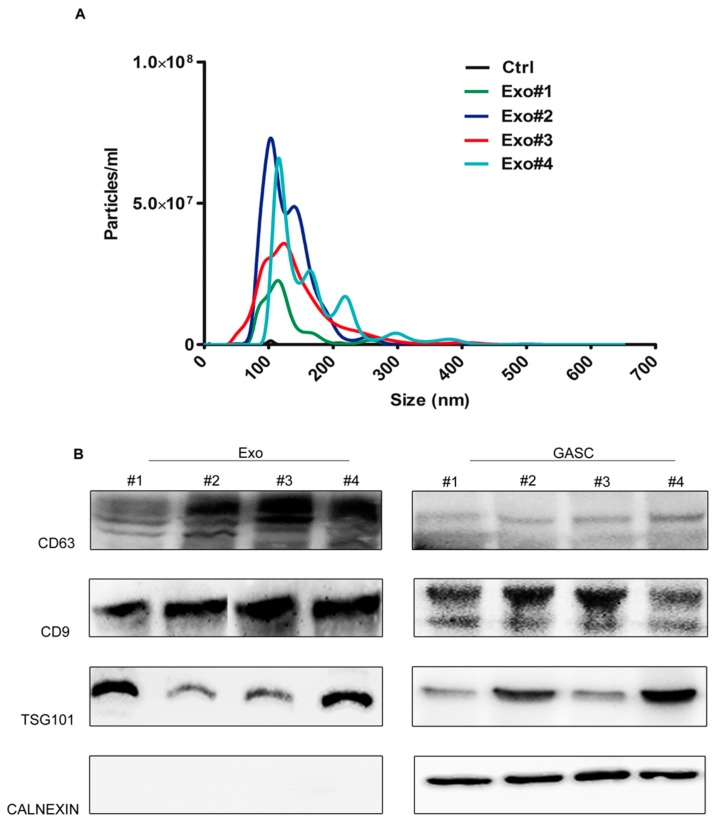
Characterisation of glioma associated stem cell (GASC)-derived exosomes. (**A**) Representative graphs of nanoparticle tracking analysis (NTA) on a preparation obtained from a nonconditioned supernatant (Ctrl) and on exosomes (Exo) isolated from supernatants conditioned for 48 h by 4 GASC cell populations (Exo#1, Exo#2, Exo#3 and Exo#4). (**B**) Western Blotting for exosome-specific markers (tetraspanins CD63, CD9 and TSG-101) and for cell endoplasmic reticulum marker (calnexin) on 4 exosomes lysates (Exo#1–4) and on paired producing cells (GASC#1–4).

Four exosomes preparations obtained from GASC conditioned medium were first examined by nanoparticle tracking analysis (NTA). Particle size ranged from 109.7 to 123.2 nm with an average diameter of 115.5 ± 5.6 nm (Figure 1A). Vesicles were not detected in non-conditioned medium used as a control. 

As assessed by Western blot, exosome preparations were enriched in CD63 and CD9, two transmembrane tetraspanins used as exosomal markers, and in TSG101 (tumour susceptibility gene 101)—a key component of the ESCRT-I complex (Endosomal Sorting Complex Required for Transport). Conversely, exosome preparations did not express Calnexin, an Endoplasmic Reticulum Protein, highly expressed in the cellular counterpart, thus excluding the presence of cellular contaminants (Figure 1B).

### 2.2. Proteomic Analysis

Protein content of exosomes from two different GASC cultures (S82 and S104) was analysed. Protein lysates were separated on Sodyum Dodecyl-Sulphate PolyAcrylamide Gel Electrophoresis SDS-PAGE and gel was cut to divide samples into six macrobands approximately corresponding to six molecular weight fractions: >130 kDa; 97–130 kDa; 66–97 kDa; 47–66 kDa; 40–47 kDa; and 26–36 kDa. Peptides obtained by in-gel trypsin digestion were analysed by liquid chromatography–tandem mass spectroscopy (LC–MS/MS).

Sequence database searches were performed using the MASCOT software (Matrixscience.com) giving a list of 316 elements (Tables S1 and S2). Four filtering steps have been applied to the list to fish out interesting candidates. (1) Significant MASCOT score: Since on average, individual ions scores > 44 indicate identity or extensive homology (*p* < 0.05), we excluded from Mascot lists proteins identified with a Mascot protein score < 44. After this step, a list of 215 digits remained (140 for S82 and 75 for S104). (2) Single digit for one protein: some proteins are present also in truncated forms and they are therefore found in different bands. All the multiple digits for the same protein were removed leaving a list of 139 elements (91 for S82 and 48 for S104). (3) Only human proteins: Mascot analyses were performed also using mammals as taxonomy. Bovine proteins corresponding to human ones and identified with the same scores were considered as putative contaminants, and therefore excluded. After these first steps a list of 85 elements, exclusively human, was left (62 in sample S82 and 23 in sample S104). (4) Proteins present in both S82- and S104-derived exosomes: comparing the two samples we found only 15 proteins present in both lists (Table 1).

### 2.3. SEMA7A Is Released on Exosomes

We focused our attention on Semaphorin-L SEMAL, also known as SEMA7A, since it has been described as a promigratory protein in different physiological conditions [25,27]. Indeed, we hypothesised that SEMA7A, carried by exosomes, could provide a promoting signal for the invasive properties of GSCs.

**Figure 2:**
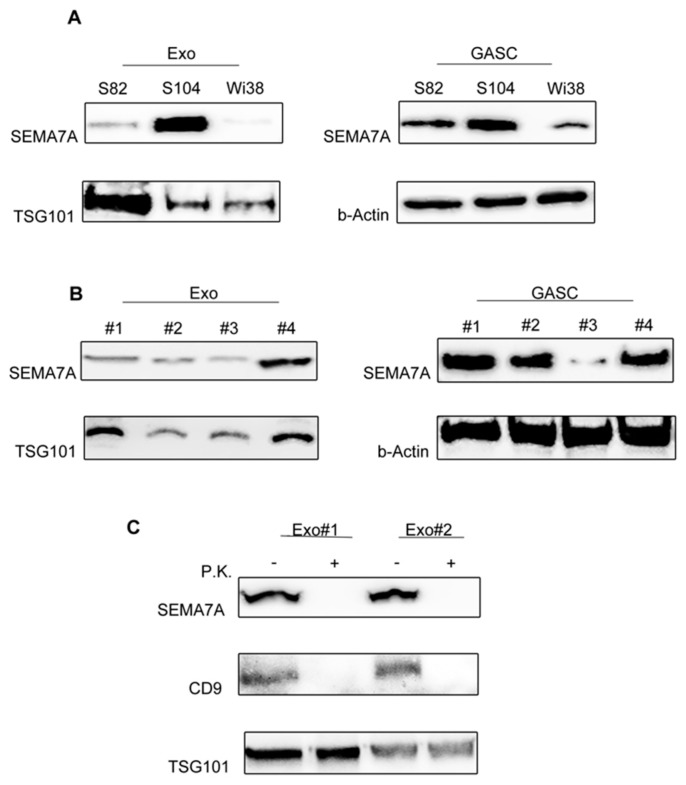
SEMA7A (Semaphorin7A) is present on the surface of Glioma Associated Stem Cells (GASC)-derived exosomes. (**A**) Western blotting showing the expression of SEMA7A in the same exosomal preparations used for the proteomic study (Exo_S82 and Exo_S104, left panel) and in the matched producing cells (GASC_S82 and GASC_S104, right panel). As healthy control, SEMA7A expression was evaluated both in Wi38 cells and in Wi38-derived exosomes (Exo-Wi38). (**B**) SEMA7A expression was assessed in 4 different GASC cell lines (GASC#1, GASC#2, GASC#3 and GASC#4) and in the respective derived exosome preparations (Exo#1, Exo#2, Exo#3 and Exo#4). Membranes were hybridised with anti-TSG101 (Tumour Susceptibility Gene 1) and beta-Actin to show the reliability of exosomes and cell lysates respectively. (**C**) Western blot analysis for SEMA7A, CD9 and TSG101 in Exo#1 and Exo#2 preparations either untreated (−) or subjected to proteinase K treatment (P.K., +).

To verify our hypothesis, we first validated the presence of SEMA7A on exosomal preparations from the producing cell lines used for the proteomic analysis (Figure 2A). Western Blot on S82 and S104 exosomal lysates and on the paired secreting cells lysates confirmed the presence of SEMA7A in both samples. We also analysed exosomes derived from a human fibroblast cell line Wi38, a mesenchymal cell line derived from nontumour tissue, as control. We observed that SEMA7A is expressed in the cells, but not released in vesicles produced by the healthy cell type, suggesting that the release of SEMA7A in exosomes is a peculiarity of mesenchymal stromal cells resident in the glioblastoma microenvironment.

The presence of SEMA7A was further confirmed in the 4 GASC cell lines and relative exosomes, subsequently used in functional experiments (Figure 2B). 

SEMA7A is a glycosylphosphatidylinositol-linked membrane protein that, once released by the cells, can be either found in the vesicular membrane or inside the membrane bilayer [30].

To elucidate where SEMA7A is accumulated in exosomes, we treated intact exosomes with Proteinase K, a broad substrate-specific endopeptidase. As showed in Figure 2C, Proteinase K completely removed SEMA7A, similarly to the multipass membrane tetraspanin CD9, while the inner TSG101 (Tumour Susceptibility Gene-101) was not affected by digestion, supporting the idea that SEMA7A is exposed on the external side of exosomes and therefore could signal directly on the target cells.

### 2.4. Expression of SEMA7A Receptors on GSC

Since SEMA7A is exposed on the surfaces of GASC-exosomes, we verified whether it could interact with receptors expressed on the GSC membrane.

SEMA7A has been shown to bind to PlexinC1 [25] and to β1-integrin receptors [31].

Plexin C1 is a member of a large family of transmembrane receptors with high affinity for semaphorins [32]. In neurons, Sema7A-PlexinC1 signalling regulates synapse development and neuroglial plasticity [33,34], while, in cancer, PlexinC1 is involved in cell migration and proliferation [35]. 

The importance of β1-integrin receptors in SEMA7A signalling has been demonstrated by Pasterkamp et al. by blocking the binding between Sema7A and its receptor, resulting in the inhibition of Sema7A-dependent neurite outgrowth in olfactory bulb neurons [31]. 

**Figure 3:**
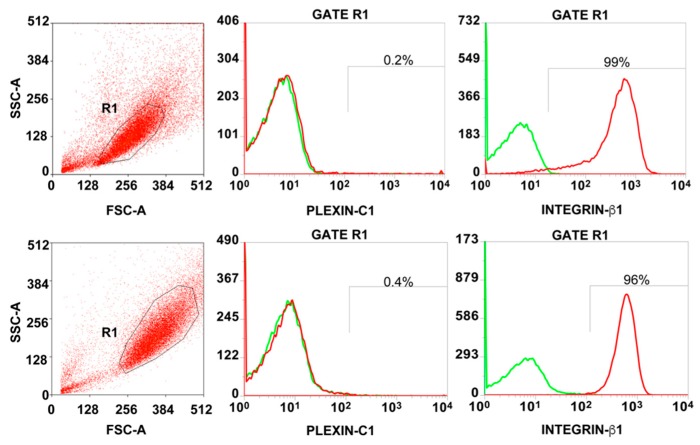
Expression of SEMA7A (Semaphorin7A) receptors on Glioma Stem Cells (GSC). Representative flow cytometry analysis showing the level of Plexin C1 and Integrin β1 on the surface of two GSC cell populations. The percentage of positive cells was calculated within the R1-Gate, representing the GSC population based on forward and side scatter (FSC/SSC) parameters. Histograms overlays show isotype control staining profile (green histograms) versus specific antibody staining profile (red histograms).

Therefore, we first verified, by flow cytometry, the presence of the SEMA7A receptors PlexinC1 and β1-integrin on the surface of GSC. As shown in Figure 3, 97.5 ± 2.12% of GSC expressed β1-integrin at high intensity, while the percentage of cells expressing PlexinC1 was almost undetectable (mean = 0.3 ± 0.14% positive cells)**.**

In light of this observation, we hypothesised that SEMA7A exposed on GASC-exosomes could signal, through its interaction with β1-integrin, on GSC.

### 2.5. SEMA7A and Exosomes Activates FAK Signalling through β1-Integrin on GSC

The most relevant pathway activated by Integrin receptors is the nonreceptor protein tyrosine kinase focal adhesion kinase (FAK), which is rapidly phosphorylated after integrin receptor binding, leading to the activation of cytoskeleton proteins responsible for integrin signal propagation [36].

**Figure 4:**
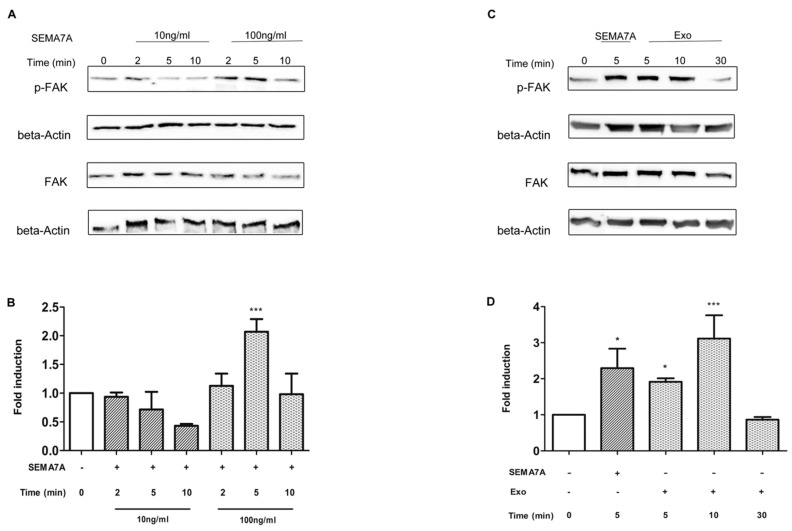
SEMA7A (Semaphorin7A) and Glioma Associated Stem Cells (GASC)-derived exosomes activate FAK signalling on glioma stem cells. (**A**,**B**) Glioma Stem Cells (GSC) were treated with either recombinant SEMA7A-Fc for 2, 5 and 10 min at 10 ng/mL or 100 ng/mL and paired GASC-derived exosomes (10 µg/mL) for 5, 10 and 30 min (**C**,**D**). Total cells lysates were resolved on 10% Sodium Dodecyl Sulphate PolyAcrylamide Gel Electrophoresis (SDS-PAGE) and blotted to identify the phosphorylated and non-phosphorylated form of focal adhesion kinase protein (FAK) (**A**,**C**). (**B**,**D**) Densitometric analysis was performed using the software available in the Gel-doc instrument (Alliance Uvitec, Ltd. Cambridge, UK) to quantify the level of FAK (Focal Adhesion Kinase) phosphorylation. Histograms represent results reported as fold change of p-FAK of treated vs. untreated cells (Ctrl). Values were calculated as the ratio of IOD (Integrated Optical Density) pFAK/FAK after normalisation on beta actin level. Data are presented as mean ± standard deviation (*n* = 4). *** *p* < 0.001 vs. Ctrl; * *p* < 0.05 vs. Ctrl. Statistical analysis was performed by repeated measurements one-way Anova followed by Bonferroni Multiple Comparison post-test (GraphPad Prism 5).

To verify whether SEMA7A activates FAK pathway, we treated GSC with exogenous recombinant SEMA7A-Fc, for 2, 5 and 10 min at the concentration of 10 ng/mL and 100 ng/mL, and assessed the level of tyrosine phosphorylation (Tyr397) of FAK (p-FAK) by Western blot (Figure 4A). As shown in Figure 4B, SEMA7A-Fc treatment increased the level of FAK phosphorylation and the peak of p-FAK was observed at 5 min with a concentration of 100 ng/mL (*p* < 0.0001 vs. untreated cells, Ctrl). Likewise, the treatment of GSC with exosomes, at a concentration of 10 μg/mL, increased the level of p-FAK after 5 and 10 min (*p* = 0.0002 vs. Ctrl cells). Conversely, maintaining exosomes for 30 min, diminished the p-FAK at levels of control (Figure 4C,D). We therefore choose the concentration of 100 ng/mL for SEMA7A-Fc and 10 μg/mL for GASC-exosome to exploit their effect on GSC motility.

### 2.6. SEMA7A and Exosomes Increases GSC Motility

**Figure 5:**
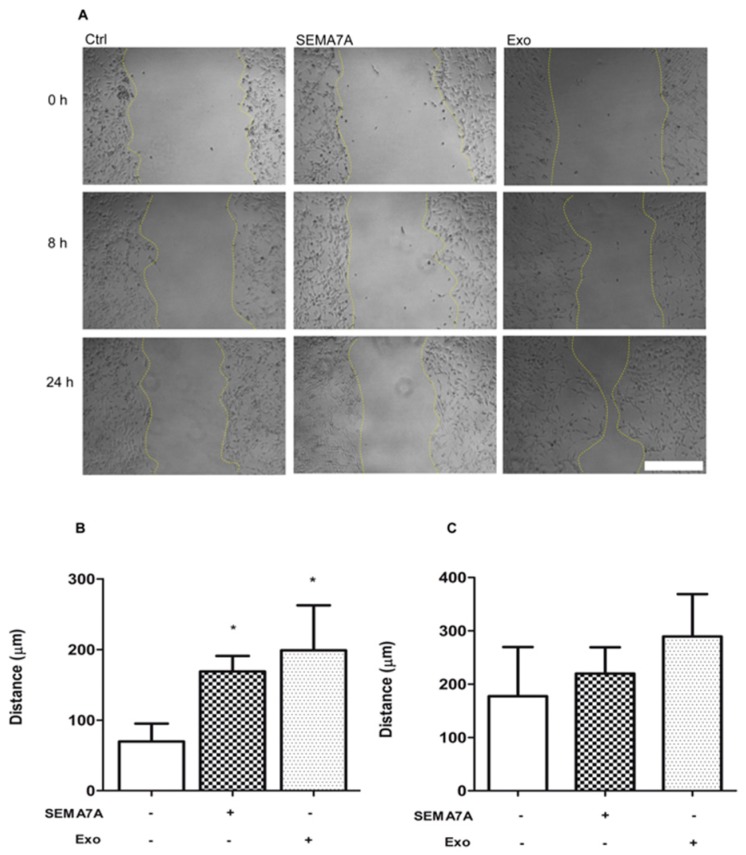
SEMA-7A (Semaphorin7A) and Glioma Associated Stem Cells (GASC)-derived exosomes stimulate motility of Glioma Stem Cells (GSC) cell populations. (**A**) Representative images of scratched GSC cultures untreated (Ctrl), exposed to SEMA-7A-Fc 100 ng/mL (SEMA7A) or GASC-derived exosomes (Exo) for 8 and 24 h. (**B**,**C**) Distance covered by cells treated as described in (A) and untreated cells (Ctrl) after 8 (B) and 24 h (C) was calculated measuring the width of the gap between the two margins of scratched monolayer. Scale bar = 200 µm. Data are presented as means ± standard deviation of 4 replicates (* *p* < 0.01 vs. Ctrl). Statistical analysis was performed by repeated measurements one-way ANOVA followed by Dunn’s Multiple Comparison post-test (GraphPad Prism 5, San Diego, California).

We performed a motility scratch assay to investigate whether SEMA7A-Fc treatment affected the migratory properties of GSC. A gap was obtained in confluent GSC by scratching the cell monolayer with a tip and the distance covered after treatment with recombinant SEMA7A-Fc (100 ng/mL) and GASC-exosomes (10 μg/mL) were measured, after 8 and 24 h. As shown in Figure 5, cells treated with SEMA7A-Fc were significantly faster in repairing the gap compared to control (covered distance in 8 h = 169 ± 21.9 µm vs. 69.62 ± 25.50 µm in SEMA7A-Fc treated and Ctrl cells, respectively). Likewise, a significant improvement in the motility of GSC was observed after treatments with exosomes (covered distance in 8 h = 198.8 ± 63. 9 µm). Thus, SEMA7A represents a promigratory signal for GSCs, and this effect is presumably mediated by FAK pathway activation.

### 2.7. Impact of SEMA7A-β1-Integrin Receptor Pathway on GSC Motility

To show that β1-integrin is the intermediate receptor between SEMA7A-exosomes and FAK in GSC, we first tested a functional blocking antibody to β1-integrin on GSC and evaluated changes of p-FAK, as described above (Figure 4). 

**Figure 6:**
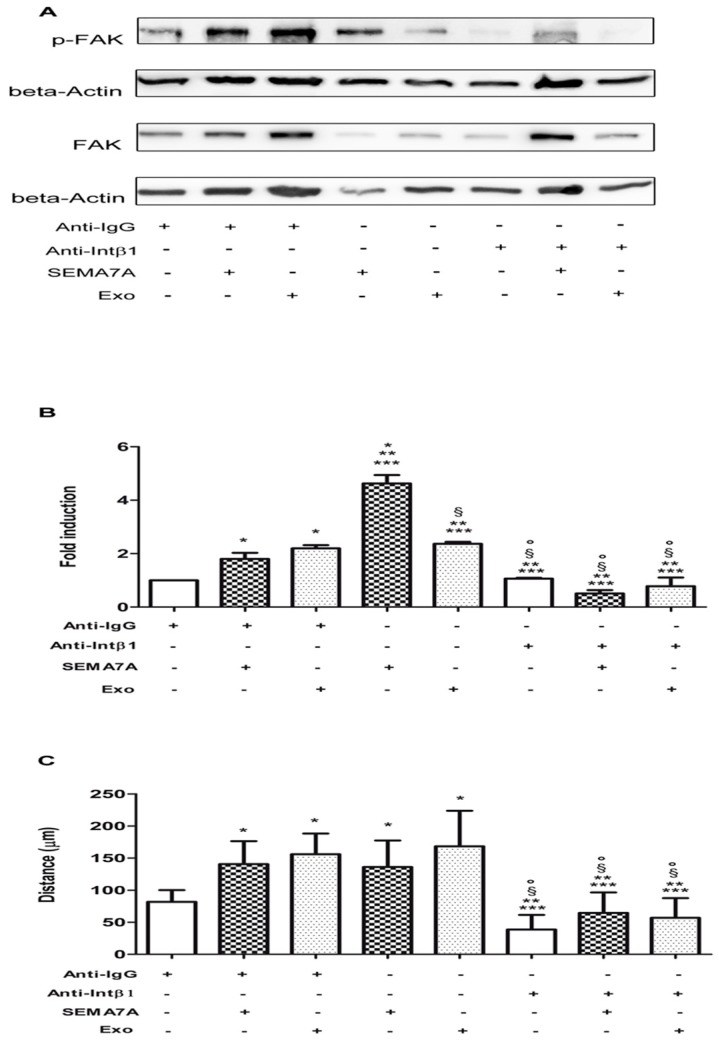
Blocking integrin beta1 on GSC (Glioma Stem Cells) affects the activation of Focal Adhesion Kinase (FAK) signalling and the motility of Glioma Stem Cells (GSC) induced by either Semaphorin7A (SEMA7A) or Glioma Associated Stem Cells (GASC)-derived exosomes. (**A**) Representative Western blotting for FAK and pFAK of GSC treated with anti-Immunoglobulin G (Anti-IgG) antibody, 5 µg/mL for 65 min, anti-Immunoglobulin G (anti-IgG)antibody, 5 µg/mL for 60 min and SEMA7A 100 ng/mL for 5 min, anti-IgG antibody 5 µg/mL for 60 min and GASC-derived exosomes 10 μg/mL for 5 min, SEMA7A 100 ng/mL for 5 min, GASC-derived exosomes 10 μg/mL for 5 min, anti-Integrin beta 1 blocking antibody 5 µg/mL for 65 min, anti-Integrin blocking antibody 5 µg/mL for 60 min and SEMA7A 100 ng/mL for 5 min and anti-Integrin blocking antibody 5 µg/mL for 60 min and GASC-derived exosomes 10 μg/mL for 5 min. (**B**) Densitometric analysis was performed using the software available in the Gel-doc instrument (Alliance Uvitec, Ltd. Cambridge, UK) to quantify the level of FAK phosphorylation. The histogram represents results reported as the Fold Change of p-FAK of different treatments vs. cells treated with the anti-IgG antibody. Values were calculated as the ratio of IOD (Integrated Optical Density) pFAK/FAK after normalisation on beta actin level. (**C**) A motility assay was performed in cells treated as described in B. Histogram represents the distance covered after 8 h, calculated measuring the width of the gap between the two margins of scratched monolayer. Data are presented as means ± standard deviation of 4 replicates. * *p* < 0.05 vs. Anti-IgG, ** *p* < 0.05 vs. Anti-IgG+SEMA-7A, *** *p* < 0.05 vs. Anti-IgG+ Exo, § *p* < 0.05 vs. SEMA-7A, ° *p* < 0.05 vs. Exo. Statistical analysis was performed by repeated measurements one-way ANOVA followed by Bonferroni Multiple Comparison post-test (GraphPad Prism 5, San Diego, California).

Figure 6A,B shows that (1) FAK was quickly phosphorylated after exposure to SEMA7A-Fc and GASC-exosomes; (2) FAK phosphorylation was abrogated when cells were treated by anti-β1-integrin antibody; and (3) FAK phosphorylation was not recovered despite the addition of SEMA7A-Fc or GASC-exosomes.

Therefore, the inactivation of β1-integrin receptor completely neutralised the activation of the FAK pathway stimulated by SEMA7A or GASC-exosomes. This effect was specific to β1 blocking antibody, since treating GSC with an unrelated antibody (anti-mouse IgG), had no effect on SEMA7A-mediated FAK phosphorylation.

To test whether the molecular interference on SEMA7A-β1-integrin-FAK might impact on GSC motility, we performed a Scratch assay to evaluate the GSC’s migratory performances, following exposure to recombinant SEMA7A-Fc alone, in combination with anti-β1-integrin blocking antibody or with anti-mouse IgG as control. Since our hypothesis is that SEMA7A transported by exosomes in the glioma microenvironment could be responsible, at least in part, of the promigratory effect on GSC, we performed a Scratch assay on GSC exposed to exosomes alone or in combination with β1-Integrin-blocking antibody. Histograms in Figure 6C clearly show that the distance covered by SEMA7A-Fc and exosomes-treated cells measured after 8 h (133.3 ± 42.26μm and 174 ± 51.23 μm, respectively), was significantly longer when compared with cells treated with anti-mouse IgG antibody (79.30 ± 20.66 μm). When the binding of SEMA7A-exosomes with β1-integrin receptor was inhibited, cells were significantly slower (37.32 ± 22.95 μm) and their migration was not restored after SEMA7A-Fc or exosomes introduction (62.07 ± 31.46 μm and 55.20 ± 30.43 μm, respectively). 

Therefore, our results strongly suggest that SEMA7A exposed on GASC-released exosomes, upon binding to β1-integrin on the surface of GSCs, accelerates their migration, which is correlated with tumour aggressiveness.

## 3. Discussion

It is well known that glioblastoma cells subvert their microenvironment from a tumour-suppressive to a tumour-supporting condition, which promotes proliferation, angiogenesis and invasion [7]. This phenomenon requires a continuous cross-talk between tumour cells and nontumour components, such as stromal cells, extracellular matrix and immune cells [37].

In our previously established in vitro model of GBM microenvironment, we found that exosomes, released by GASC, have a tumour-supporting role towards GSC [19].

Therefore, we performed a descriptive proteomic analysis of exosomes released by GASC, to understand which components could be involved in the GASC tumour-supporting function.

Results from mass spectrometry revealed 15 proteins present in both exosomes preparations analysed. Overall, all the proteins found in this study could contribute to the tumour-supporting function of GASC’ exosomes. Fibronectin1 FBN1, TIMP metalloprotease inhibitor 1)(TIMP1) and Phospholipid transfer protein (PLTP) were described as promoter of metastasis, resistance to apoptosis and migratory properties in ovarian cancer, melanoma and glioma, respectively [38,39,40]; Collagen Alpha-1 (VI) chain (COL6A1) and Collagen Alpha-2 (VI) chain (COL6A2) are involved in the guidance of neural crest cells, during CNS development [41] and in the motoneuron axon growth [42]; plasminogen activator inhibitor-1 (PAI-1), is a well-known marker overexpressed in several forms of cancer [43]; Galectin-3-binding protein promotes integrin-mediated cell adhesion [44] and has been found elevated in the serum of patients with cancer [45], although not present in Exocarta, it has been described in the exosomes of ovarian carcinoma cells [46] and it has been suggested to be an important component of tumour microenvironment [47]; Serotransferrin (transferrin) is an abundant blood plasma glycoprotein whose main function is to bind and transport iron throughout the body. Interestingly, Carlsson et al., 2013 [48] showed that ~5% of human serotransferrin glycoforms bind galectin-3 and are targeted to a different endocytic pathway and that the galectin-3-bound glycoform is increased in cancer. Although eight out of 15 proteins have been previously described in exosomes of some sources, five of them are completely new. Among these, we focused our attention on SEMA7A, because of its role in physiological and pathological conditions. One of the first study showed that SEMA7A has a function on the immune system, being a potent activator of monocytes, stimulating their chemotaxis and production of inflammatory cytokines [49]. Pasterkamp et al. were the first to describe SEMA7A as a guidance signal responsible to stimulate axon outgrowth, during the formation of lateral olfactory tracts [31]. The authors showed that SEMA7A binds to integrin beta1 receptors and activates the mitogen activated protein kinase pathway (MAPK). Moreover, the use of a beta-1 integrin inhibitory antibody blocked the neurite outgrowth. Moreover, SEMA7A is expressed in the secretome of U87 glioblastoma cells at higher level than in less aggressive and invasive cell lines (T98, U118) [50].

In summary SEMA7A can be regarded as a promigratory stimulus and since motility of glioma cells is one of the properties responsible for the infiltrative nature of GBM, we hypothesised that SEMA7A, released by GASC through exosomes, can act as one of the messages stimulating GSC motility/migration.

We confirmed both the presence of SEMA7A on four exosome preparations and, most importantly, its exposure on the external surface of exosomes. On the other hand, cytofluorimetric analysis revealed the presence of integrin beta 1, but not Plexin C1—two major SEMA7A receptors—on glioma stem cells (GSCs). This suggested that SEMA7A could directly signal to GSC through integrin beta1.

Integrins represent the major cellular receptors for extracellular matrix involved in the regulation of cell migration through their coupling with cytoskeletal and signalling molecules, clustering in focal adhesion in adherent cells and Focal adhesion kinase (FAK) has been established as a key component of signal transduction triggered by integrins [51]. Autophosphorylation and activation of FAK lead to modulation of cytoskeletal proteins, cytoskeletal reorganisation and force generation [52,53].

Regulation of cell migration by integrin signalling through FAK leading to cancer pathogenesis and aggressiveness has been assessed in many cell types [54]. β1 integrins have been implicated in brain invasion of glioma cells in animal models using antisense RNA to reduce integrin expression [55].

In our in vitro model, relying on GASC and GSC obtained from the same tumour, the treatment of GSC, both with recombinant SEMA7A-Fc and exosomes produced by GASC, stimulated a rapid FAK phosphorylation and significantly increased the motility of GSC. Using an antibody blocking β1-integrin receptors, we observed a reduction of FAK activation at the level of the control and a decreased speed of GSC, in covering the gap generated in the motility assay. Concomitant addition of SEMA7AFc or GASC-exosomes to anti-integrin beta1 antibody was not able to rescue FAK engagement or motility of GSC.

The involvement of SEMA7A-integrin β1 in cell migration and tumour invasiveness is not completely new: Black et al. identified a high expression of SEMA7A in ductal in situ breast cancer characterised by poor prognosis and distant metastases, and showed the involvement of SEMA7A in promoting tumour cell invasion and lymphangiogenesis, via activation of β1-integrin [29].

Moreover, other members of the semaphorin family were identified in exosomes of the glioblastoma microenvironment. SEMA3A released in GSC’ exosomes disrupt the endothelial barrier, thus promoting the vascular permeability and invasion in the surrounding brain parenchyma [56].

The novelty of our work is to indicate, for the first time, the interaction SEMA7A-β1-integrin as a new mediator in the cross-talk occurring in the glioma microenvironment between exosomes produced by glioma stromal cells and GSC, becoming an interesting new possible therapeutic target 

In fact, integrin ligand binding and regulatory sites are externally exposed and made them good accessible drug candidates [57]. Indeed, many inhibitors have been developed. Of the number of clinical trials started, some reached late stage and some inhibitors have even launched for treatment. Of the Intβ1 inhibitors evaluated in the clinical trials, a pan β1 monoclonal antibody P5 (claimed to predominantly act on α5β1) is reported to enhance cisplatin efficacy in lung adenocarcinoma cells and is in use in phase 3 trial for non-small cell lung cancer [58].

In the light of our result it would be interesting to further investigate the possibility to reduce tumour infiltration by the use of β1 inhibitors like the P5 antibody. 

Altogether our results give new insights on how stromal cells in the glioblastoma microenvironment could contribute to the increased aggressiveness of the tumour.

## 4. Materials and Methods

### 4.1. Isolation and Culture of GASC and GSC

Cells were isolated from patients affected by a de novo supratentorial glioblastoma (GBM). All patients, not previously treated, underwent surgical resection of the tumour at the Neurosurgery Department of the Udine Hospital. The independent ethic committee of the Azienda Ospedaliero-Universitaria of Udine has approved the research (Consent 102/2011/Sper, 02 August, 2011 and Consent 196/2014/Em, 03 December, 2014). Written informed consents were obtained from all patients and clinical investigations have been conducted according to the Declaration of Helsinki. GASC were isolated from 6 tumour samples, as previously described [19]. In 4 cases, GSCs from the same tumour samples were cultured [59] in order to have 4 GASC-GSC pairs. See Appendix A.

### 4.2. Isolation of Exosomes 

Cells were cultured in expansion medium for three passages and then seeded at 6000 cells/cm^2^ in 100 mm Petri dishes. After 24 h, expansion medium was replaced with a serum–linoleic acid bovine serum albumin-depleted medium. Cells were maintained until 70–80% confluence (48 h). Wi38 cells were cultured in Dulbecco’s Modified Eagle Medium D-MEM+10% exosome-depleted foetal bovine serum. Exosomes were isolated from the collected supernatants using ExoQuick-TC Exosome precipitation solution (System Biosciences, Palo Alto, California), according to manufacturer’s protocol. The exosomal pellets were resuspended in phosphate-buffered saline (PBS) or radio immunoprecipitation assay (RIPA) buffer; see Appendix A.

### 4.3. Nanoparticle Tracking Analysis

Concentration and particle size of purified exosomes were measured by Nanosight (LM10, Malvern system Ltd., U.K.), equipped with a 405 nm laser. Briefly, each sample, once properly diluted, was recorded for 60 s with a detection threshold set at maximum. Temperature was monitored throughout the measurements. Vesicle size distribution and an estimated concentration of NTA (Nanoparticle Tracking Analysis) profiles were obtained from the given raw data files.

### 4.4. Proteomic Analyses

For proteomic analysis, 30 µg of total exosomal proteins obtained from two different GASC cultures (S82 and S104) were used. Protein lysates were separated on SDS-PAGE (Gel Tris-Glycine 10%) and the gel was cut to divide samples into six macrobands and treated essentially as previously described [60]. Fragments obtained by in-gel trypsin digestion were analysed by LC–MS/MS with ionic trap, on an Agilent 1200 series nanoHPLC interfaced to an HCTultra IT (Bruker Daltonics, Billerica, Massachussetts). Peptide masses and MS/MS spectra were exported as ‘mgf’ files and database search was performed with the MASCOT MS/MS (Mascot-Peptide mass finger-print) Ion Search option). See Appendix A.

### 4.5. Western Blot

Whole-cell and exosomes extract proteins were obtained by lysis in RIPA buffer. Thirty micrograms of proteins was resolved on SDS-PAGE, transferred and immobilised on a 0.45 μm nitrocellulose membrane (Amersham, London, UK).

Membranes were incubated overnight at 4 °C, with goat polyclonal to CD63 (LSBio, Seattle, Washington) mouse monoclonal to CD9 (Clone Ts9) (Thermo Fisher, Walthman, Massachussetts) mouse monoclonal to TSG101 (Clone 4A10) (Abcam, Cambridge, UK), rabbit polyclonal to Calnexin (Abcam rabbit polyclonal to SEMA7A (Novus Biologicals, Colorado, US) and rabbit polyclonal to beta-Actin (Sigma-Aldrich, Italy). Primary antibodies were detected using horseradish peroxidase-linked secondary antibodies (DAKO, Cambridge, UK) and visualised using the enhanced chemiluminescent detection system (SuperSignal West Dura Extended Duration Substrate, Thermo Scientific, Walthman, Massachussetts); see Appendix A.

### 4.6. Proteinase K Treatment of GASC-Exosomes

30 µg of GASC-derived exosomes resuspended in PBS were incubated with 100 μg/mL Proteinase K (Sigma-Aldrich, Italy) or the same volume of water as control, for 1 h, at 37 °C, with regular shaking. Proteinase K was inhibited with phenylmethylsulfonyl fluoride PMSF 5 mM treatment for 10 min at 72 °C. Exosomes were then lysed and analysed by western blot.

### 4.7. Flow Cytometry

Proliferating GSC were detached with TRYPLE Express (Gibco Walthman, Massachussetts) and incubated with conjugated primary antibody anti-integrin beta 1 (CD29-APC, BD-Biosciences) or unconjugated goat anti-human PlexinC1 (Invitrogen, Carlsband, California). Plexin C1 was revealed using Alexa-Fluor 647 donkey anti-goat secondary antibody (Jackson ImmunoResearch, Cambridge, UK). Plexin C1 expression was previously evaluated on human CD14+ monocytes (positive control, Figure S1), as detailed in Appendix A. Proper conjugated isotype matched antibodies were used as negative control. 

At least 2 × 10^4^ events were collected by FACS CANTO II (Becton Dickinson (BD)-Biosciences, US) and analysed using Summit Software (Dako Cytomation, Denmark).

### 4.8. Evaluation of FAK Phosphorylation (Tyr-397) after SEMA7A and GASC-Exosome Stimulation or Integrin Beta1 Blocking on GSC

GSC were seeded at 1 × 10^4^/cm^2^ in complete Neurobasal-A medium. After 24 h, cells were treated with Human Recombinant SEMA7A Fc Chimera (R&D System, Minneapolis, Minnesota) for 2, 5 or 10 min at 10 or 100 ng/mL or untreated.

For treatments with GASC-exosomes, GSCs were exposed to 10 µg/mL of exosomes for 5, 10 and 30 min. For treatments with anti-integrin beta1 blocking antibody, GSC were treated as detailed in Appendix A.

Cells were washed in cold PBS, immediately harvested in RIPA buffer, at 4 °C, and proteins were extracted as previously described. Twenty micrograms of proteins was resolved on 10% SDS/PAGE, and then transferred and immobilised on a 0.45 μm nitrocellulose membrane, (Amersham).

The membranes were incubated with rabbit-anti human to FAK antibody, rabbit-anti human to phospho-FAK (Tyr-397) antibody (Cell Signalling Technology, Danvers, Massachusetts) and with rabbit polyclonal to beta-Actin. Levels of FAK and p-FAK were evaluated by densiometric analysis, using ImageJ. IOD (Integrated Optical Density) was analysed for each condition and Fold Change of p-FAK was calculated vs. untreated (Ctrl) cells, after normalisation on β -actin expression.

### 4.9. Scratch Assay

GSC were seeded on 48-well plates at 3 × 10^4^ cell/cm^2^. At confluence, the cell monolayer was straight-scraped with a p10 pipette tip. Cellular debris was removed and culture medium was replaced with fresh one containing the treatments described in Appendix A. Each experimental condition was performed four-fold. Images were acquired at 8 h and 24 h after treatments, by a Leica DMI 6000B microscope connected to a Leica DFC350FX camera (10× objective, numerical aperture 0.25, Wetzlar, Germany). Images were then compared and quantified by Image J.

## 5. Conclusions

In this study, we described for the first time SEMA7A-integrin β1 interaction as a new mediator of the cross-talk occurring between microenvironment and glioma stem cells, through the release of exosomes. This interaction could be regarded as a new therapeutic target in the GBM field.

## Figures and Tables

**Table 1:** List of 15 human proteins shared by two GASC-derived exosome preparations analysed by proteomics (Exo_S82, Exo_S104). Seven proteins were already described in Exocarta database and shown in the right column is a list of sources of exosomes in which they were observed. In bold are highlighted the six proteins not previously referenced in Exocarta.

Accession Number	Protein	Exocarta	Origin of Exosomes
**XP_005257115.2**	“collagen alpha-1(I) chain isoform X2 [*Homo sapiens*]”		
**CO6A1_HUMAN**	“Collagen alpha-1(VI) chain OS = *Homo sapiens* OX = 9606 GN = COL6A1 PE = 1 SV = 3”	1291	hepatocellular carcinoma; melanoma, nasopharyngeal carcinoma, prostate cancer cells; saliva; urina
**CO6A2_HUMAN**	“Collagen alpha-2(VI) chain OS = *Homo sapiens* OX = 9606 GN = COL6A2 PE = 1 SV = 4”	1292	hepatocellular carcinoma; saliva
**FBN1_HUMAN**	“Fibrillin-1 OS = *Homo sapiens* OX = 9606 GN = FBN1 PE = 1 SV = 3”	2200	endothelial cells
**NP_002017.1**	“fibronectin isoform 3 pre-proprotein [*Homo sapiens*]”		
**LG3BP_HUMAN**	“Galectin-3-binding protein OS = *Homo sapiens* OX = 9606 GN = LGALS3BP PE = 1 SV = 1”		ovarian cancer
**G3P_HUMAN**	“Glyceraldehyde-3-phosphate dehydrogenase OS = *Homo sapiens* OX = 9606 GN = GAPDH PE = 1 SV = 3”	2597	bladder, breast cancer; colorectal cancer; hepatocellular carcinoma, intestinal epithelial, melanoma, mesothelioma, neuroblastoma ovarian cancer, prostate cancer, squamous carcinoma tracheobronchial, trabecular meshwork cells; aqueous humour; hepatocytes; keratinocytes; platelets; saliva; urine.
**LDHA_HUMAN**	“L-lactate dehydrogenase A chain OS = *Homo sapiens* OX = 9606 GN = LDHA PE = 1 SV = 2”	3939	bladder, breast cancer; colorectal cancer; hepatocellular carcinoma, intestinal epithelial, melanoma, nasopharyngeal carcinoma, ovarian cancer, prostate cancer, squamous carcinoma cells; hepatocytes; platelets; thymus; urine
**TIMP1_HUMAN**	“Metalloproteinase inhibitor 1 OS = *Homo sapiens* OX = 9606 GN = TIMP1 PE = 1 SV = 1”	7076	keratinocytes; nasopharyngeal carcinoma cells; squamous carcinoma cells
**PLTP_HUMAN**	“Phospholipid transfer protein OS = *Homo sapiens* OX = 9606 GN = PLTP PE = 1 SV = 1”	5360	keratinocytes; platelets; prostate cancer cells; saliva; squamous carcinoma cells
**PAI1_HUMAN**	“Plasminogen activator inhibitor 1 OS = *Homo sapiens* OX = 9606 GN = SERPINE1 PE = 1 SV = 1”		
**AAC34741.1**	“semaphorin L partial [*Homo sapiens*]”		
**TRFE_HUMAN**	“Serotransferrin OS = *Homo sapiens* OX = 9606 GN = TF PE = 1 SV = 3”		
**QSOX1_HUMAN**	“Sulfhydryl oxidase 1 OS = *Homo sapiens* OX = 9606 GN = QSOX1 PE = 1 SV = 3”		
**SJM31191.1**	“Sulfotransferase [*Homo sapiens*]”		

## Supplementary Materials

The following are available online at https://www.mdpi.com/2072-6694/11/6/758/s1, Figure S1: Representative Flow Cytometry analysis showing the level of Plexin C1 on the surface of human monocytes. Table S1: Proteins extracted from 30 µg exosomes derived from the sample S-82 and identified by LC–MS/MS analyses. Table S2: Proteins extracted from 30 µg exosomes derived from the sample S-104 and identified by LC–MS/MS analyses.

## Appendix A

### Appendix A.1. Methods

#### Appendix A.1.1. Isolation and Culture of Glioma-Associated Stem Cells and Glioma Stem Cells

Glioblastoma (GBM, grade IV glioma) fragments were first disaggregated mechanically with scalpels, and then enzymatically dissociated in a 0.025% Collagenase Type II solution (Worthington, UK, ) in Joklik Modified Eagle’s Medium (Sigma-Aldrich, Italy) for 5 min at 37 °C. Collagenase activity was then neutralised by the addition of 10% Fetal Bovine Serum in Joklik modified Eagle’s Medium (Sigma-Aldrich, Italy). Cell suspension was centrifuged at 500 *g* for 10 min and filtered through a sieve (BD Falcon, US) in order to select a population less than 40 μm in diameter. Two-million freshly isolated human cells were plated onto 100 mm diameter, human fibronectin (Sigma-Aldrich, Italy)-coated dishes (BD Falcon), in an expansion medium composed as follows; 60% low glucose Dulbecco’s Modified Eagle’s Medium (DMEM, Gibco, Walthman, Massachussetts), 40% MCDB-201, 1mg/mL linoleic acid-BSA, 10^−9^ M dexamethasone, 10^−4^ M ascorbic acid-2 phosphate, 1X insulin-transferrin-sodium selenite (all from Sigma-Aldrich, Italy), 2% fetal bovine serum (StemCell Technologies, Mexico), 10 ng/mL human PDGF-BB and 10 ng/mL human EGF (both from Peprotech EC). GSC were isolated from the same patients’ biopsy using the aforementioned protocol, but seeded at a density of 2 × 10^4^ cells/cm^2^ onto laminin (Laminin from Engelbreth-Holm-Swarm murine sarcoma basement membrane, Sigma-Aldrich, Italy)-coated dishes in a growing serum-free medium composed as follows; Neurobasal-A medium (Gibco Walthman, Massachussetts), 2 mM L-glutamine (Sigma-Aldrich, Italy), 1X N2 supplement (Gibco Walthman, Massachussetts), 25 μg/mL Insulin, Penicillin-streptomycin, 100 μg/mL human apo-trasferrin (Sigma-Aldrich, Italy), 1× B-27 supplement (Gibco, Walthman, Massachussetts), 20 ng/mL h-FGF-basic (Peprotech, UK) and 20 ng/mL h-EGF (Peprotech, UK). Both cell populations were cultured in an incubator at 37 °C, 5% O_2_/5% CO_2_. Half medium was replaced with fresh one every 4 days. Once cells reached 70 ± 80% of confluence, they were detached with TrypLE Express (Invitrogen, Carlsband, California) and re-plated at a density of 3 × 10^3^/cm^2^ and 1 × 10^4^/cm^2^ (GASC and GSC, respectively).

#### Appendix A.1.2. Isolation of Exosomes

Supernatants were collected after 48 h conditioning, centrifuged at 3000 *g* for 30 min at 4 °C to remove cells and debris and filtered through a 0.2 μm filter to remove particles larger than 200 nm. Supernatants were then concentrated using centrifugal filter units (Amico-Ultra15, Merck Millipore, Burlington, Massachussetts) in regenerated cellulose membranes, with pore size of 100 kDa, by centrifuge at 4700 *g* for 20 min at 4 °C. The ultrafiltered supernatants were then incubated with ExoQuick-TC Exosome precipitation solution (System Biosciences, Palo Alto, California) in a 5:1 dilution overnight at 4 °C and centrifuged at 1500 *g*, 15 min at 4 °C. Exosomal pellets were dissolved in PBS or RIPA buffer (NaCl 150 mM, 25 mM Tris-HCl, pH 7.6, 1% IGEPAL, (Octylphenoxy poly(ethyleneoxy)ethanol), 0.1% SDS, 1% SodiumDeoxycholate, 1 mM dithiothreitol, 1 mM Na_2_ VO_4_, 1 mM Sodium Fluoride, 0.5 mM Phenylmethanesulfonyl fluoride, Protease Inhibitor Cocktail), based on future applications.

### Appendix A.2. Proteomic Analyses

#### Appendix A.2.1. In-gel Digestion and LC–MS/MS Analyses

Proteins extracted from exosomes were separated on 10% SDS polyacrylamide gel, stained with R250 Coomassie Brilliant Blue (CBB) and destained with 10% acetic acid. Electrophoretic lanes were cut into six equivalent parts—>130 kDa, 97–130 kDa, 66–97 kDa, 47–66 kDa, 40–47 kDa and 26–36 kDa—and processed essentially as previously described (40). LC–MS/MS analyses were performed on an Agilent 1200 series nanoHPLC (High Performance Liquid Chromatography) interfaced to an HCTultra IT (Bruker Daltonics, Billerica, Massachussetts). 20 out of 30 µL resuspended peptides were loaded at a flow rate of 15 µL/min on a trap column (300SB–C18 USP L1 0.3 × 5 mm, 5 µm, Agilent) for 5 min with 98% H2O, 2% ACN and 0.1% TFA. The trap column was then connected to the analytical column (Zorbax Nano RR 300SB–C18 0.075 × 150 mm, 3.5 µm) and the peptides eluted with a linear H2O/ACN (Acetonitrile) gradient (Solvent A: H2O 0.1% HCOOH, Solvent B: ACN 0.1% HCOOH; 60 min gradient from 2 to 60% Solvent B; flow: 200 nL/min). Eluted peptides were analysed online with an HCTultra IT (Bruker Daltonics, Billerica, Massachussetts) using a nanoelectrospray interface. IT control was performed by the Esquire Control software (version 6.1, Bruker Daltonics, Billerica, Massachussetts). LC–MS/MS analyses were elaborated with the Data Analysis software (version 3.4, Bruker Daltonics, Billerica, Massachussetts ). Peptide masses and MS/MS spectra were exported as ‘.mgf’ files and database search was performed with the MASCOT MS/MS Ion Search option: database: NCBIprot and SwissProt; taxonomy: *Homo sapiens* (human); enzyme: trypsin; allowing up to three instances of missed cleavage; Fixed modification: none; variable modification: none; peptide tol.: 1.2 Da; MS/MS tol.: 0.6 Da; peptide charge: 2 and 3; mass: monoisotopic; Instrument: ESI-TRAP. Mascot analyses were performed also using Mammalia (mammals) as taxonomy. Bovine proteins corresponding to human ones and identified with the same scores were considered as putative contaminants and evidenced in red within the reported tables. Since on average, individual ions scores >44 indicate identity or extensive homology (*p* < 0.05) we excluded from Mascot lists proteins identified with only one peptide and with a Mascot protein score <44. Raw MS data (.mgf) are available upon request.

#### Appendix A.2.2. Isolation and Staining of CD14 Positive Monocytes from Human Blood Samples

CD14 positive monocytes were used as positive control to evaluate the expression of PlexinC1. To isolate CD14+ cells, whole blood samples from healthy donors were collected in EDTA (ethylene diamine tetra-acetic acid) -tubes by the Department of Transfusion Medicine (University Hospital of Udine) after a written informed consent was signed. PBMCs were separated by centrifugation at 700 *g* for 20 min on a Ficoll Hypaque density gradient (Millipore, Burlington, Massachussetts) and resuspended at 1 × 10^6^ cells/mL in 0,5% bovine serum albumin (BSA), 2 mM EDTA in phosphate buffered saline (PBS), pH 7.2. CD14+ cells were purified from PBMCS (Peripheral Blood Mononuclear Cells) after immuno-magnetic labelling with CD14 Microbeads (MACS-Miltenyi Biotec, Bergisch Gladbach, Germany) by positive selection in a magnetic field, following manufacturer’s suggestion (Miltenyi Biotec, Bergisch Gladbach, Germany). Cells were double-stained with conjugated primary antibody to CD14-APC (BD-Biosciences, US) and unconjugated goat anti-human to PlexinC1 (Invitrogen, Carlsband, California ) at 0.2 µg/10^4^ cells. Plexin C1 was revealed using Alexa-Fluor 647 donkey anti-goat secondary antibody (Jackson ImmunoResearch, Cambridge, UK), diluted 1:800. Properly conjugated isotype matched antibodies were used as negative control.

#### Appendix A.2.3. Western Blot

Whole-cell extract proteins were obtained by cells’ lysis in RIPA Buffer for 40 min on ice and centrifugation at 10.000 *g* for 15 min at 4 °C, and then the protein-enriched supernatants were collected. The extraction of proteins from exosomes was performed using the above described RIPA buffer, for 30 min at 4 °C. Proteins concentration was measured by BCA protein assay (Euroclone, Italy).

30 μg of proteins were resolved on SDS-PAGE, transferred and immobilised on a 0.45 μm nitrocellulose membrane (Amersham, London, UK).

Membranes were blocked by incubation for 1 h at room temperature, with 5% non-fat milk or 5% Bovine Serum Albumin in TBS (Tris-HCl 50 mM, pH 7.4, NaCl 150 mM) containing 0.2% Tween 20 and hybridised, overnight at 4 °C, with goat polyclonal to CD63 (LSBio, Seattle, Washington) diluted 1:500, mouse monoclonal to CD9 (Clone Ts9) (Thermo Fisher, Walthman, Massachussetts) diluted 1:1000, mouse monoclonal to TSG101 (Clone 4A10) (Abcam, Cambridge, UK), diluted 1:1000, rabbit polyclonal to Calnexin (Abcam, Cambridge, UK), diluted 1:1000, rabbit polyclonal to SEMA7A (Novus Biologicals, Colorado, US), diluted 1:1000 and rabbit polyclonal to beta-Actin (Sigma-Aldrich, Italy), diluted 1:2000. Primary antibodies were detected using horseradish peroxidase-linked anti-mouse, anti-rabbit or anti-goat secondary antibodies (DAKO, Cambridge, UK) and visualised using the enhanced chemiluminescent detection system (SuperSignal West Dura Extended Duration Substrate, Thermo Scientific, Walthman, Massachussetts).

### Appendix A.3. Evaluation of FAK Phosphorylation (Tyr-397) after SEMA7A and GASC-Exosomes Stimulation or Integrin Beta1 Blocking on GSCs

For treatments with anti-integrin beta1 blocking antibody, GSCs were seeded at 1 × 10^4^/cm^2^ in complete Neurobasal-A medium. After 24 h, cells were treated by

1. 100 ng/mL of Sema7A-Fc (R&D System), for 5 min
2. 10 µg/mL of GASC-exosomes, for 5 min
3. 5 µg/mL of Anti-IgG antibody (Millipore), for 65 min
4. 5 µg/mL of Anti-IgG antibody for 60 min, +100 ng/mL of Sema7A-Fc, for 5 min
5. 5 µg/mL of Anti-IgG antibody for 60 min, +10 µg/mL of GASC-exosomes, for 5 min
6. 5 µg/mL of Mouse monoclonal to integrin beta1 [P5D2] (Abcam, ab24693), for 65 min
7. 5 µg/mL of Mouse monoclonal to integrin beta1 for 60 min, +100 ng/mL of Sema7A-Fc, for 5 min;
8. 5 µg/mL of Mouse monoclonal to integrin beta1, for 60 min, +10 µg/mL of GASC-exosomes, for 5 min.

All treatments were performed in a 5% O_2_/5% CO_2_ incubator at 37 °C, and repeated using four GSC cell populations and four paired GASC-exosomes preparations.

Cells were washed in cold PBS, immediately harvested in RIPA buffer at 4 °C, and proteins were extracted as previously described. Twenty micrograms of proteins was resolved on 10% SDS/PAGE, and then transferred and immobilised on a 0.45 μm nitrocellulose membrane, (Amersham).

The membranes were processed as described in the “Western Blot” section, incubated with rabbit-anti human to FAK antibody and with rabbit-anti human to phospho-FAK (Tyr-397) antibody (Cell Signalling Technology, Danver, Massachussetts) diluted 1:1000 in 5% Bovine Serum Albumin in TBS. Levels of FAK and p-FAK expression were evaluated by densitometric analysis, using ImageJ. IOD (Integrated Optical Density) was analysed for each condition and pFAK/FAK ratio was calculated, after normalisation on β-actin expression.
