# Supplementary material for: Semaphorin-7A on Exosomes: A Promigratory Signal in the Glioma Microenvironment
Source: Cancers (Basel). 2019 May 30;11(6):758. doi: 10.3390/cancers11060758 (PMC6628148; doi:10.3390/cancers11060758)
Supplement: Supplementary file 1 [file cancers-11-00758-s001.pdf]

Supplementary materials

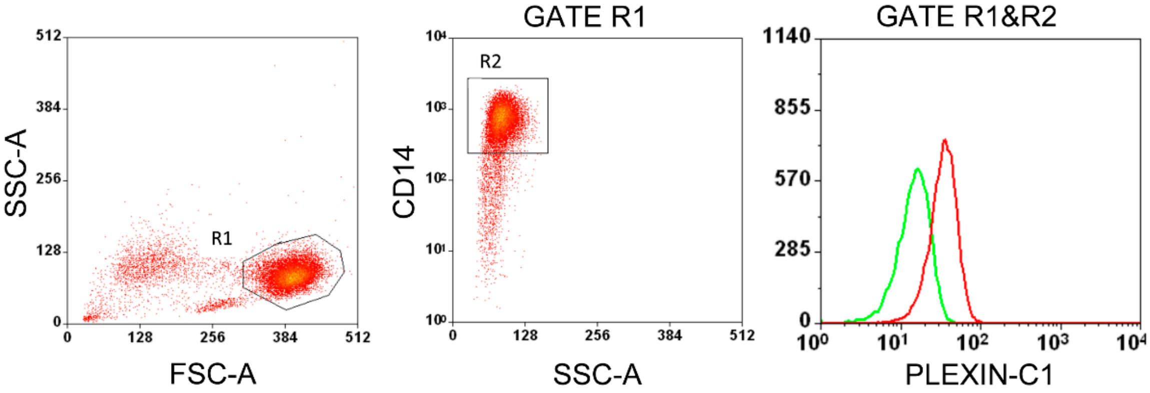

Figure S1. Representative Flow Cytometry analysis showing the level of Plexin C1 on the surface of human monocytes.

Table S1. Proteins extracted from 30 µg exosomes derived from the sample S-82 and identified by LC–MS/MS analyses.

F	DB	Accession	S	N M	S82–band 1–Description
1	N	NP_002017.1	1987	66	"fibronectin isoform 3 preproprotein [Homo sapiens]"
2	S	PGS1_HUMAN	630	19	"Biglycan OS=Homo sapiens OX=9606 GN=BGN PE=1 SV=2"
2	N	EAW72861.1	510	17	"biglycan isoform CRA_a [Homo sapiens]"
3	S	CO6A1_HUMAN	473	15	"Collagen alpha-1(VI) chain OS=Homo sapiens OX=9606 GN=COL6A1 PE=1 SV=3"
4	S	TARSH_HUMAN	362	10	"Target of Nesh-SH3 OS=Homo sapiens OX=9606 GN=ABI3BP PE=1 SV=1"
5	N	AAB94054.3	333	12	"pro alpha 1(I) collagen [Homo sapiens]"
5	N	AAA51995.1	282	11	"alpha 1 (I) chain propeptide partial [Homo sapiens]"
6	N	CAH56139.1	273	7	"hypothetical protein partial [Homo sapiens]"
7	S	A2MG_HUMAN	153	4	"Alpha-2-macroglobulin OS=Homo sapiens OX=9606 GN=A2M PE=1 SV=3"
8	S	ALBU_HUMAN	124	4	"Serum albumin OS=Homo sapiens OX=9606 GN=ALB PE=1 SV=2"
9	S	LAMC1_HUMAN	102	2	"Laminin subunit gamma-1 OS=Homo sapiens OX=9606 GN=LAMC1 PE=1 SV=3"
10	N	CAA23761.1	100	2	"unnamed protein product partial [Homo sapiens]"
11	S	FLNB_HUMAN	88	1	"Filamin-B OS=Homo sapiens OX=9606 GN=FLNB PE=1 SV=2"
12	S	VIME_HUMAN	83	1	"Vimentin OS=Homo sapiens OX=9606 GN=VIM PE=1 SV=4"
13	S	LAMB1_HUMAN	67	1	"Laminin subunit beta-1 OS=Homo sapiens OX=9606 GN=LAMB1 PE=1 SV=2"
14	S	HEMO_HUM	60	3	"Hemopexin OS=Homo sapiens OX=9606 GN=HPX PE=1 SV=2"

		AN			
15	S	TRFE_HUMAN	55	2	"Serotransferrin OS=Homo sapiens OX=9606 GN=TF PE=1 SV=3"
16	S	GPC1_HUMAN	53	2	"Glypican-1 OS=Homo sapiens OX=9606 GN=GPC1 PE=1 SV=2"
17	S	PTX3_HUMAN	49	1	"Pentraxin-related protein PTX3 OS=Homo sapiens OX=9606 GN=PTX3 PE=1 SV=3"
18	S	AMD_HUMAN	47	1	"Peptidyl-glycine alpha-amidating monooxygenase OS=Homo sapiens OX=9606 GN=PAM PE=1 SV=2"
19	N	SJM31191.1	46	3	"Sulfotransferase [Homo sapiens]"
20	N	AAA52002.1	46	1	"alpha-1 type III collagen partial [Homo sapiens]"
21	N	AAA53376.1	41	1	"fibronectin partial [Homo sapiens]"
22	S	TLN1_HUMAN	32	1	"Talin-1 OS=Homo sapiens OX=9606 GN=TLN1 PE=1 SV=3"
23	N	AKD42826.1	29	1	"anti-HIV-1 immunoglobulin light chain variable region partial [Homo sapiens]"
24	S	PLTP_HUMAN	25	1	"Phospholipid transfer protein OS=Homo sapiens OX=9606 GN=PLTP PE=1 SV=1"
25	S	UAP1L_HUMAN	19	1	"UDP-N-acetylhexosamine pyrophosphorylase-like protein 1 OS=Homo sapiens OX=9606 GN=UAP1L1 PE=1 SV=2"
**F**	**DB**	**Accession**	**S**	**NM**	**S82–band 2–Description**
1	S	CO6A1_HUMAN	2520	81	"Collagen alpha-1(VI) chain OS=Homo sapiens OX=9606 GN=COL6A1 PE=1 SV=3"
2	N	XP_005257115.2	720	19	"collagen alpha-1(I) chain isoform X2 [Homo sapiens]"
3	N	CAA23761.1	356	13	"unnamed protein product partial [Homo sapiens]"
4	S	CO6A2_HUMAN	311	12	"Collagen alpha-2(VI) chain OS=Homo sapiens OX=9606 GN=COL6A2 PE=1 SV=4"
5	N	NP_002017.1	191	5	"fibronectin isoform 3 preproprotein [Homo sapiens]"
6	N	XP_024301816.1	157	3	"alpha-mannosidase 2 isoform X2 [Homo sapiens]"
7	S	PGS1_HUMAN	122	3	"Biglycan OS=Homo sapiens OX=9606 GN=BGN PE=1 SV=2"
8	S	VINC_HUMAN	121	3	"Vinculin OS=Homo sapiens OX=9606 GN=VCL PE=1 SV=4"
9	S	SPTN1_HUMAN	92	3	"Spectrin alpha chain non-erythrocytic 1 OS=Homo sapiens OX=9606 GN=SPTAN1 PE=1 SV=3"
10	S	ALBU_HUMAN	89	3	"Serum albumin OS=Homo sapiens OX=9606 GN=ALB PE=1 SV=2"
11	S	PLTP_HUMAN	73	1	"Phospholipid transfer protein OS=Homo sapiens OX=9606 GN=PLTP PE=1 SV=1"
12	S	ACTN1_HUMAN	65	1	"Alpha-actinin-1 OS=Homo sapiens OX=9606 GN=ACTN1 PE=1 SV=2"

13	S	IPO5_HUMAN	64	1	"Importin-5 OS=Homo sapiens OX=9606 GN=IPO5 PE=1 SV=4"
14	N	AAB28324.1	49	1	"beta-fodrin partial [Homo sapiens]"
15	N	AAA52016.1	48	1	"complement H factor partial [Homo sapiens]"
16	S	A4_HUMAN	48	1	"Amyloid-beta A4 protein OS=Homo sapiens OX=9606 GN=APP PE=1 SV=3"
17	N	SJM28484.1	47	1	"Adenosylhomocysteinase [Homo sapiens]"
18	S	TRFE_HUMAN	47	1	"Serotransferrin OS=Homo sapiens OX=9606 GN=TF PE=1 SV=3"
19	S	A2MG_HUMAN	41	1	"Alpha-2-macroglobulin OS=Homo sapiens OX=9606 GN=A2M PE=1 SV=3"
20	S	ENPP2_HUMAN	36	1	"Ectonucleotide pyrophosphatase/phosphodiesterase family member 2 OS=Homo sapiens OX=9606 GN=ENPP2 PE=1 SV=3"
21	N	AAA52002.1	35	1	"alpha-1 type III collagen partial [Homo sapiens]"
22	N	SJM35999.1	33	1	"Trehalose/maltose import ATP-binding protein MalK [Homo sapiens]"
23	S	CSTN1_HUMAN	31	1	"Calsyntenin-1 OS=Homo sapiens OX=9606 GN=CLSTN1 PE=1 SV=1"
24	S	PROM2_HUMAN	30	1	"Prominin-2 OS=Homo sapiens OX=9606 GN=PROM2 PE=1 SV=1"
25	N	CAE45795.1	28	1	"hypothetical protein partial [Homo sapiens]"
26	S	AMPQ_HUMAN	24	1	"Aminopeptidase Q OS=Homo sapiens OX=9606 GN=LVRN PE=1 SV=4"
27	N	SJM31191.1	24	1	"Sulfotransferase [Homo sapiens]"
28	N	XP_024308106.1	23	1	"uracil phosphoribosyltransferase homolog isoform X1 [Homo sapiens]"
**F**	**DB**	**Accession**	**S**	**NM**	**S82–band 3–Description**
1	S	TRFE_HUMAN	741	31	"Serotransferrin OS=Homo sapiens OX=9606 GN=TF PE=1 SV=3"
2	S	QSOX1_HUMAN	468	15	"Sulfhydryl oxidase 1 OS=Homo sapiens OX=9606 GN=QSOX1 PE=1 SV=3"
3	S	CO6A1_HUMAN	419	14	"Collagen alpha-1(VI) chain OS=Homo sapiens OX=9606 GN=COL6A1 PE=1 SV=3"
4	S	ACTN4_HUMAN	403	12	"Alpha-actinin-4 OS=Homo sapiens OX=9606 GN=ACTN4 PE=1 SV=2"
4	N	ACJ24535.1	333	9	"actinin alpha 1 isoform 3 [Homo sapiens]"
5	S	LG3BP_HUMAN	387	23	"Galectin-3-binding protein OS=Homo sapiens OX=9606 GN=LGALS3BP PE=1 SV=1"
6	S	PLTP_HUMAN	229	7	"Phospholipid transfer protein OS=Homo sapiens OX=9606 GN=PLTP PE=1 SV=1"
7	S	SCG2_HUMAN	214	10	"Secretogranin-2 OS=Homo sapiens OX=9606 GN=SCG2 PE=1 SV=2"
8	S	BIP_HUMAN	166	4	"Endoplasmic reticulum chaperone BiP OS=Homo sapiens OX=9606 GN=HSPA5 PE=1 SV=2"

9	N	AAC34741.1	153	5	"semaphorin L partial [Homo sapiens]"
10	N	XP_005257115.2	151	4	"collagen alpha-1(I) chain isoform X2 [Homo sapiens]"
11	S	LUM_HUMAN	140	2	"Lumican OS=Homo sapiens OX=9606 GN=LUM PE=1 SV=2"
12	S	C1S_HUMAN	130	2	"Complement C1s subcomponent OS=Homo sapiens OX=9606 GN=C1S PE=1 SV=1"
13	N	CAA23761.1	126	3	"unnamed protein product partial [Homo sapiens]"
14	N	AAH23006.2	115	5	"HSP90AA1 protein partial [Homo sapiens]"
15	S	PGS1_HUMAN	111	2	"Biglycan OS=Homo sapiens OX=9606 GN=BGN PE=1 SV=2"
16	S	ALBU_HUMAN	97	4	"Serum albumin OS=Homo sapiens OX=9606 GN=ALB PE=1 SV=2"
17	S	MOES_HUMAN	96	4	"Moesin OS=Homo sapiens OX=9606 GN=MSN PE=1 SV=3"
18	S	PTX3_HUMAN	74	1	"Pentraxin-related protein PTX3 OS=Homo sapiens OX=9606 GN=PTX3 PE=1 SV=3"
19	S	CO6A2_HUMAN	66	2	"Collagen alpha-2(VI) chain OS=Homo sapiens OX=9606 GN=COL6A2 PE=1 SV=4"
20	S	FINC_HUMAN	60	2	"Fibronectin OS=Homo sapiens OX=9606 GN=FN1 PE=1 SV=4"
21	S	C1R_HUMAN	57	2	"Complement C1r subcomponent OS=Homo sapiens OX=9606 GN=C1R PE=1 SV=2"
22	N	EAW68718.1	54	2	"hemopexin isoform CRA_a [Homo sapiens]"
23	N	AAB05933.1	50	2	"extracellular matrix protein 1 [Homo sapiens]"
24	S	BGH3_HUMAN	51	1	"Transforming growth factor-beta-induced protein ig-h3 OS=Homo sapiens OX=9606 GN=TGFBI PE=1 SV=1"
25	S	EF2_HUMAN	50	1	"Elongation factor 2 OS=Homo sapiens OX=9606 GN=EEF2 PE=1 SV=4"
26	S	EKI1_HUMAN	46	1	"Ethanolamine kinase 1 OS=Homo sapiens OX=9606 GN=ETNK1 PE=1 SV=1"
27	S	ENPL_HUMAN	42	1	"Endoplasmin OS=Homo sapiens OX=9606 GN=HSP90B1 PE=1 SV=1"
28	S	PLOD1_HUMAN	40	2	"Procollagen-lysine2-oxoglutarate 5-dioxygenase 1 OS=Homo sapiens OX=9606 GN=PLOD1 PE=1 SV=2"
29	N	ACE74969.1	38	1	"immunoglobulin heavy chain variable region partial [Homo sapiens]"
30	S	FBLN1_HUMAN	37	1	"Fibulin-1 OS=Homo sapiens OX=9606 GN=FBLN1 PE=1 SV=4"
31	N	SJM31191.1	37	1	"Sulfotransferase [Homo sapiens]"
32	S	PTK7_HUMAN	28	1	"Inactive tyrosine-protein kinase 7 OS=Homo sapiens OX=9606 GN=PTK7 PE=1 SV=2"
33	N	EAW60825.1	24	1	"signal transducer and activator of transcription 3 (acute-phase response factor) isoform CRA_f [Homo sapiens]"
34	S	MEF2A_HUMAN	19	1	"Myocyte-specific enhancer factor 2A OS=Homo sapiens OX=9606 GN=MEF2A PE=1 SV=1"
**F**	**DB**	**Accession**	**S**	**NM**	**S82–band 4–Description**

1	S	BGH3_HUMAN	675	20	"Transforming growth factor-beta-induced protein ig-h3 OS=Homo sapiens OX=9606 GN=TGFBI PE=1 SV=1"
2	S	ALBU_HUMAN	462	18	"Serum albumin OS=Homo sapiens OX=9606 GN=ALB PE=1 SV=2"
3	S	CO6A1_HUMAN	309	11	"Collagen alpha-1(VI) chain OS=Homo sapiens OX=9606 GN=COL6A1 PE=1 SV=3"
4	S	NUCB1_HUMAN	284	10	"Nucleobindin-1 OS=Homo sapiens OX=9606 GN=NUCB1 PE=1 SV=4"
5	S	HEMO_HUMAN	241	9	"Hemopexin OS=Homo sapiens OX=9606 GN=HPX PE=1 SV=2"
6	S	MMP2_HUMAN	189	5	"72 kDa type IV collagenase OS=Homo sapiens OX=9606 GN=MMP2 PE=1 SV=2"
7	S	LUM_HUMAN	147	3	"Lumican OS=Homo sapiens OX=9606 GN=LUM PE=1 SV=2"
8	S	TRFE_HUMAN	146	5	"Serotransferrin OS=Homo sapiens OX=9606 GN=TF PE=1 SV=3"
9	N	BAG64863.1	136	3	"unnamed protein product [Homo sapiens]"
10	N	CAA23761.1	125	3	"unnamed protein product partial [Homo sapiens]"
11	S	MOES_HUMAN	90	3	"Moesin OS=Homo sapiens OX=9606 GN=MSN PE=1 SV=3"
12	N	NP_000079.2	90	2	"collagen alpha-1(I) chain preproprotein [Homo sapiens]"
13	N	BAH11999.1	86	1	"unnamed protein product [Homo sapiens]"
14	N	SJM31191.1	81	8	"Sulfotransferase [Homo sapiens]"
15	S	SAP_HUMAN	71	1	"Prosaposin OS=Homo sapiens OX=9606 GN=PSAP PE=1 SV=2"
16	S	KPYM_HUMAN	69	1	"Pyruvate kinase PKM OS=Homo sapiens OX=9606 GN=PKM PE=1 SV=4"
17	S	SCG2_HUMAN	68	3	"Secretogranin-2 OS=Homo sapiens OX=9606 GN=SCG2 PE=1 SV=2"
18	S	PDIA3_HUMAN	65	2	"Protein disulfide-isomerase A3 OS=Homo sapiens OX=9606 GN=PDIA3 PE=1 SV=4"
19	S	ISLR_HUMAN	52	2	"Immunoglobulin superfamily containing leucine-rich repeat protein OS=Homo sapiens OX=9606 GN=ISLR PE=2 SV=1"
20	S	CO3A1_HUMAN	47	1	"Collagen alpha-1(III) chain OS=Homo sapiens OX=9606 GN=COL3A1 PE=1 SV=4"
21	S	OLFL3_HUMAN	45	2	"Olfactomedin-like protein 3 OS=Homo sapiens OX=9606 GN=OLFML3 PE=2 SV=1"
22	S	TSSK4_HUMAN	41	1	"Testis-specific serine/threonine-protein kinase 4 OS=Homo sapiens OX=9606 GN=TSSK4 PE=1 SV=1"
23	S	CH60_HUMAN	41	1	"60 kDa heat shock protein mitochondrial OS=Homo sapiens OX=9606 GN=HSPD1 PE=1 SV=2"
24	S	PTX3_HUMAN	37	1	"Pentraxin-related protein PTX3 OS=Homo sapiens OX=9606 GN=PTX3 PE=1 SV=3"
25	S	ANT3_HUMAN	36	1	"Antithrombin-III OS=Homo sapiens OX=9606 GN=SERPINC1 PE=1 SV=1"

26	N	NP_001333184.1	31	1	"ubiquitin carboxyl-terminal hydrolase 28 isoform f [Homo sapiens]"
27	S	PLTP_HUMAN	30	1	"Phospholipid transfer protein OS=Homo sapiens OX=9606 GN=PLTP PE=1 SV=1"
28	N	AAH21119.1	29	1	"GAPVD1 protein [Homo sapiens]"
29	N	AAQ62468.1	28	1	"thioredoxin reductase 1 partial [Homo sapiens]"
30	N	AAA52018.1	25	1	"chromogranin A [Homo sapiens]"
31	S	CAD23_HUMAN	25	1	"Cadherin-23 OS=Homo sapiens OX=9606 GN=CDH23 PE=1 SV=2"
32	S	MCPH1_HUMAN	24	1	"Microcephalin OS=Homo sapiens OX=9606 GN=MCPH1 PE=1 SV=4"
33	S	CD109_HUMAN	23	1	"CD109 antigen OS=Homo sapiens OX=9606 GN=CD109 PE=1 SV=2"
34	S	DYN3_HUMAN	22	1	"Dynamin-3 OS=Homo sapiens OX=9606 GN=DNM3 PE=1 SV=4"
35	N	SJM33335.1	20	1	"Cytochrome P450 [Homo sapiens]"
36	S	CO3_HUMAN	20	1	"Complement C3 OS=Homo sapiens OX=9606 GN=C3 PE=1 SV=2"
**F**	**DB**	**Accession**	**S**	**NM**	**S82–band 5–Description**
1	S	PAI1_HUMAN	1624	44	"Plasminogen activator inhibitor 1 OS=Homo sapiens OX=9606 GN=SERPINE1 PE=1 SV=1"
2	S	ACTB_HUMAN	350	16	"Actin cytoplasmic 1 OS=Homo sapiens OX=9606 GN=ACTB PE=1 SV=1"
3	S	PTX3_HUMAN	304	8	"Pentraxin-related protein PTX3 OS=Homo sapiens OX=9606 GN=PTX3 PE=1 SV=3"
4	N	NP_000079.2	245	6	"collagen alpha-1(I) chain preproprotein [Homo sapiens]"
5	S	ISLR_HUMAN	220	6	"Immunoglobulin superfamily containing leucine-rich repeat protein OS=Homo sapiens OX=9606 GN=ISLR PE=2 SV=1"
6	S	TRFE_HUMAN	197	7	"Serotransferrin OS=Homo sapiens OX=9606 GN=TF PE=1 SV=3"
7	S	ENOA_HUMAN	183	3	"Alpha-enolase OS=Homo sapiens OX=9606 GN=ENO1 PE=1 SV=2"
8	S	ALBU_HUMAN	178	5	"Serum albumin OS=Homo sapiens OX=9606 GN=ALB PE=1 SV=2"
9	S	HEMO_HUMAN	167	6	"Hemopexin OS=Homo sapiens OX=9606 GN=HPX PE=1 SV=2"
10	S	SPRC_HUMAN	140	7	"SPARC OS=Homo sapiens OX=9606 GN=SPARC PE=1 SV=1"
11	S	ALDOA_HUMAN	132	4	"Fructose-bisphosphate aldolase A OS=Homo sapiens OX=9606 GN=ALDOA PE=1 SV=2"
12	S	CO6A1_HUMAN	121	3	"Collagen alpha-1(VI) chain OS=Homo sapiens OX=9606 GN=COL6A1 PE=1 SV=3"
13	S	VIME_HUMA	117	4	"Vimentin OS=Homo sapiens OX=9606 GN=VIM PE=1 SV=4"

		N			
13	N	AAA61281.2	112	4	"vimentin partial [Homo sapiens]"
14	N	CAA23761.1	109	4	"unnamed protein product partial [Homo sapiens]"
15	N	AAA52002.1	104	2	"alpha-1 type III collagen partial [Homo sapiens]"
16	S	FSTL1_HUMAN	92	3	"Follistatin-related protein 1 OS=Homo sapiens OX=9606 GN=FSTL1 PE=1 SV=1"
17	N	AAX62798.1	82	2	"aberrant LSLCL [Homo sapiens]"
18	S	PCOC1_HUMAN	76	3	"Procollagen C-endopeptidase enhancer 1 OS=Homo sapiens OX=9606 GN=PCOLCE PE=1 SV=2"
19	S	FINC_HUMAN	72	1	"Fibronectin OS=Homo sapiens OX=9606 GN=FN1 PE=1 SV=4"
20	S	TGFB2_HUMAN	65	1	"Transforming growth factor beta-2 OS=Homo sapiens OX=9606 GN=TGFB2 PE=1 SV=1"
21	S	SPI2_HUMAN	63	3	"Serpin I2 OS=Homo sapiens OX=9606 GN=SERPINI2 PE=1 SV=1"
22	S	CBPA4_HUMAN	51	1	"Carboxypeptidase A4 OS=Homo sapiens OX=9606 GN=CPA4 PE=1 SV=2"
23	S	LRC40_HUMAN	36	1	"Leucine-rich repeat-containing protein 40 OS=Homo sapiens OX=9606 GN=LRRC40 PE=1 SV=1"
24	N	CAD38944.1	35	1	"hypothetical protein partial [Homo sapiens]"
25	N	XP_016875684.1	31	1	"ubiquitin-protein ligase E3B isoform X5 [Homo sapiens]"
26	S	MMP1_HUMAN	23	1	"Interstitial collagenase OS=Homo sapiens OX=9606 GN=MMP1 PE=1 SV=3"
27	S	CATL1_HUMAN	22	1	"Cathepsin L1 OS=Homo sapiens OX=9606 GN=CTSL PE=1 SV=2"
28	N	BAG52708.1	21	1	"unnamed protein product [Homo sapiens]"
29	N	ADE73599.1	21	1	"MHC class I antigen partial [Homo sapiens]"
30	N	EAX10114.1	20	1	"chromosome 20 open reading frame 3 isoform CRA_b [Homo sapiens]"
31	S	EPC1_HUMAN	19	1	"Enhancer of polycomb homolog 1 OS=Homo sapiens OX=9606 GN=EPC1 PE=1 SV=1"
**F**	**DB**	**Accession**	**S**	**NM**	**S82–band 6–Description**
1	N	CAA39142.1	1843	58	"type I collagen partial [Homo sapiens]"
2	N	AAA51995.1	1518	50	"alpha 1 (I) chain propeptide partial [Homo sapiens]"
3	S	CO3A1_HUMAN	411	10	"Collagen alpha-1(III) chain OS=Homo sapiens OX=9606 GN=COL3A1 PE=1 SV=4"
4	S	APOE_HUMAN	298	9	"Apolipoprotein E OS=Homo sapiens OX=9606 GN=APOE PE=1 SV=1"
5	N	BAA01989.1	239	5	"human non-muscle myosin heavy chain partial [Homo sapiens]"
6	S	TPM4_HUMAN	215	7	"Tropomyosin alpha-4 chain OS=Homo sapiens OX=9606 GN=TPM4 PE=1 SV=3"
6	N	AAL84570.1	150	4	"TPMsk3 partial [Homo sapiens]"

7	N	NP_001158887.1	206	4	"L-lactate dehydrogenase A chain isoform 4 [Homo sapiens]"
8	S	CO6A1_HUMAN	201	6	"Collagen alpha-1(VI) chain OS=Homo sapiens OX=9606 GN=COL6A1 PE=1 SV=3"
9	S	CO5A1_HUMAN	179	7	"Collagen alpha-1(V) chain OS=Homo sapiens OX=9606 GN=COL5A1 PE=1 SV=3"
9	N	NP_001265003.1	155	6	"collagen alpha-1(V) chain isoform 2 preproprotein [Homo sapiens]"
10	S	ALDOA_HUMAN	155	4	"Fructose-bisphosphate aldolase A OS=Homo sapiens OX=9606 GN=ALDOA PE=1 SV=2"
11	S	LDHB_HUMAN	152	4	"L-lactate dehydrogenase B chain OS=Homo sapiens OX=9606 GN=LDHB PE=1 SV=2"
12	S	IBP6_HUMAN	147	4	"Insulin-like growth factor-binding protein 6 OS=Homo sapiens OX=9606 GN=IGFBP6 PE=1SV=1"
13	S	CLUS_HUMAN	138	4	"Clusterin OS=Homo sapiens OX=9606 GN=CLU PE=1 SV=1"
14	S	IBP7_HUMAN	135	5	"Insulin-like growth factor-binding protein 7 OS=Homo sapiens OX=9606 GN=IGFBP7 PE=1 SV=1"
15	S	TIMP1_HUMAN	121	2	"Metalloproteinase inhibitor 1 OS=Homo sapiens OX=9606 GN=TIMP1 PE=1 SV=1"
16	S	SODE_HUMAN	121	2	"Extracellular superoxide dismutase [Cu-Zn] OS=Homo sapiens OX=9606 GN=SOD3 PE=1 SV=2"
17	S	FBN1_HUMAN	116	4	"Fibrillin-1 OS=Homo sapiens OX=9606 GN=FBN1 PE=1 SV=3"
18	S	ALBU_HUMAN	94	3	"Serum albumin OS=Homo sapiens OX=9606 GN=ALB PE=1 SV=2"
19	N	BAG60757.1	93	2	"unnamed protein product [Homo sapiens]"
20	S	STC1_HUMAN	88	1	"Stanniocalcin-1 OS=Homo sapiens OX=9606 GN=STC1 PE=1 SV=1"
21	S	VIME_HUMAN	87	2	"Vimentin OS=Homo sapiens OX=9606 GN=VIM PE=1 SV=4"
22	S	ITIH4_HUMAN	71	1	"Inter-alpha-trypsin inhibitor heavy chain H4 OS=Homo sapiens OX=9606 GN=ITIH4 PE=1 SV=4"
23	S	EXTL2_HUMAN	65	1	"Exostosin-like 2 OS=Homo sapiens OX=9606 GN=EXTL2 PE=1 SV=1"
24	S	HEMO_HUMAN	56	2	"Hemopexin OS=Homo sapiens OX=9606 GN=HPX PE=1 SV=2"
25	S	G3P_HUMAN	52	1	"Glyceraldehyde-3-phosphate dehydrogenase OS=Homo sapiens OX=9606 GN=GAPDH PE=1 SV=3"
26	S	CTHR1_HUMAN	49	1	"Collagen triple helix repeat-containing protein 1 OS=Homo sapiens OX=9606 GN=CTHRC1 PE=1 SV=1"
27	S	RLA0L_HUMAN	48	1	"60S acidic ribosomal protein P0-like OS=Homo sapiens OX=9606 GN=RPLP0P6 PE=5 SV=1"
28	S	GXLT1_HUMA	43	1	"Glucoside xylosyltransferase 1 OS=Homo sapiens OX=9606 GN=GXYLT1 PE=1 SV=2"

		N			
29	N	AAH28145.1	38	1	"THBS1 protein partial [Homo sapiens]"
30	S	ALDOC_HUMAN	38	1	"Fructose-bisphosphate aldolase C OS=Homo sapiens OX=9606 GN=ALDOC PE=1 SV=2"
31	S	FINC_HUMAN	37	1	"Fibronectin OS=Homo sapiens OX=9606 GN=FN1 PE=1 SV=4"
32	N	SJM31191.1	37	1	"Sulfotransferase [Homo sapiens]"
33	S	PAI1_HUMAN	35	1	"Plasminogen activator inhibitor 1 OS=Homo sapiens OX=9606 GN=SERPINE1 PE=1 SV=1"
34	S	TRFE_HUMAN	33	1	"Serotransferrin OS=Homo sapiens OX=9606 GN=TF PE=1 SV=3"
35	S	PRS23_HUMAN	32	1	"Serine protease 23 OS=Homo sapiens OX=9606 GN=PRSS23 PE=1 SV=1"
36	S	GTPB2_HUMAN	21	1	"GTP-binding protein 2 OS=Homo sapiens OX=9606 GN=GTPBP2 PE=1 SV=1"

LC-MS/MS: Liquid Chromatography Mass Spectrometry.

Table S2. Proteins extracted from 30 µg exosomes derived from the sample S-104 and identified by LC–MS/MS analyses.

F	DB	Accession	S	NM	S104–band 1–Description
1	N	NP_997647.1	1629	54	"fibronectin isoform 1 precursor [Homo sapiens]"
2	S	ALBU_HUMAN	294	14	"Serum albumin OS=Homo sapiens OX=9606 GN=ALB PE=1 SV=2"
3	S	A2MG_HUMAN	160	7	"Alpha-2-macroglobulin OS=Homo sapiens OX=9606 GN=A2M PE=1 SV=3"
4	S	PGS1_HUMAN	157	6	"Biglycan OS=Homo sapiens OX=9606 GN=BGN PE=1 SV=2"
5	N	SJM31191.1	89	7	"Sulfotransferase [Homo sapiens]"
6	S	TRFE_HUMAN	65	2	"Serotransferrin OS=Homo sapiens OX=9606 GN=TF PE=1 SV=3"
7	S	CERU_HUMAN	43	1	"Ceruloplasmin OS=Homo sapiens OX=9606 GN=CP PE=1 SV=1"
8	N	SJM28737.1	40	1	"Cytosine deaminase [Homo sapiens]"
9	N	EAW62750.1	19	1	"hCG2041930 partial [Homo sapiens]"
**F**	**DB**	**Accession**	**S**	**NM**	**S104–band 2–Description**
1	S	CO6A1_HUMAN	705	20	"Collagen alpha-1(VI) chain OS=Homo sapiens OX=9606 GN=COL6A1 PE=1 SV=3"
2	N	XP_005257115.2	509	16	"collagen alpha-1(I) chain isoform X2 [Homo sapiens]"
3	S	ALBU_HUMAN	198	8	"Serum albumin OS=Homo sapiens OX=9606 GN=ALB PE=1 SV=2"
4	N	NP_002017.1	164	9	"fibronectin isoform 3 preproprotein [Homo sapiens]"

5	N	CAA23761.1	152	6	"unnamed protein product partial [Homo sapiens]"
6	S	PGS1_HUMAN	147	3	"Biglycan OS=Homo sapiens OX=9606 GN=BGN PE=1 SV=2"
7	S	A2MG_HUMAN	102	4	"Alpha-2-macroglobulin OS=Homo sapiens OX=9606 GN=A2M PE=1 SV=3"
8	S	IPO5_HUMAN	75	1	"Importin-5 OS=Homo sapiens OX=9606 GN=IPO5 PE=1 SV=4"
9	S	TRFE_HUMAN	74	2	"Serotransferrin OS=Homo sapiens OX=9606 GN=TF PE=1 SV=3"
10	S	TLN1_HUMAN	70	1	"Talin-1 OS=Homo sapiens OX=9606 GN=TLN1 PE=1 SV=3"
11	S	PXDN_HUMAN	62	2	"Peroxidasin homolog OS=Homo sapiens OX=9606 GN=PXDN PE=1 SV=2"
12	S	FLNA_HUMAN	58	1	"Filamin-A OS=Homo sapiens OX=9606 GN=FLNA PE=1 SV=4"
13	S	CO6A2_HUMAN	58	2	"Collagen alpha-2(VI) chain OS=Homo sapiens OX=9606 GN=COL6A2 PE=1 SV=4"
14	N	EAW68718.1	52	2	"hemopexin isoform CRA_a [Homo sapiens]"
15	S	VINC_HUMAN	42	1	"Vinculin OS=Homo sapiens OX=9606 GN=VCL PE=1 SV=4"
16	N	SJM31191.1	40	3	"Sulfotransferase [Homo sapiens]"
17	S	STPG1_HUMAN	31	1	"O(6)-methylguanine-induced apoptosis 2 OS=Homo sapiens OX=9606 GN=STPG1 PE=1 SV=1"
18	N	CAE45795.1	25	1	"hypothetical protein partial [Homo sapiens]"
19	N	AMZ80337.1	25	1	"SHLP2 [Homo sapiens]"
20	N	ADX66002.1	18	1	"immunoglobulin variable region partial [Homo sapiens]"
**F**	**DB**	**Accession**	**S**	**NM**	**S104–band 3–Description**
1	S	TRFE_HUMAN	649	26	"Serotransferrin OS=Homo sapiens OX=9606 GN=TF PE=1 SV=3"
2	S	ACTN1_HUMAN	355	10	"Alpha-actinin-1 OS=Homo sapiens OX=9606 GN=ACTN1 PE=1 SV=2"
3	S	LG3BP_HUMAN	315	16	"Galectin-3-binding protein OS=Homo sapiens OX=9606 GN=LGALS3BP PE=1 SV=1"
4	S	ALBU_HUMAN	205	7	"Serum albumin OS=Homo sapiens OX=9606 GN=ALB PE=1 SV=2"
5	S	HS90A_HUMAN	176	8	"Heat shock protein HSP 90-alpha OS=Homo sapiens OX=9606 GN=HSP90AA1 PE=1 SV=5"
6	N	AAH49849.1	135	3	"MYH9 protein partial [Homo sapiens]"
7	S	PLOD1_HUMAN	101	2	"Procollagen-lysine 2-oxoglutarate 5-dioxygenase 1 OS=Homo sapiens OX=9606 GN=PLOD1 PE=1 SV=2"
8	N	AAC34741.1	88	2	"semaphorin L partial [Homo sapiens]"
9	S	QSOX1_HUMAN	86	2	"Sulfhydryl oxidase 1 OS=Homo sapiens OX=9606 GN=QSOX1 PE=1 SV=3"
10	S	PGS1_HUMAN	79	1	"Biglycan OS=Homo sapiens OX=9606 GN=BGN PE=1 SV=2"

11	N	BAG36698.1	68	1	"unnamed protein product [Homo sapiens]"
12	S	PLTP_HUMAN	60	1	"Phospholipid transfer protein OS=Homo sapiens OX=9606 GN=PLTP PE=1 SV=1"
13	N	SJM31191.1	53	3	"Sulfotransferase [Homo sapiens]"
14	S	FETA_HUMAN	48	2	"Alpha-fetoprotein OS=Homo sapiens OX=9606 GN=AFP PE=1 SV=1"
15	N	CAA23761.1	43	1	"unnamed protein product partial [Homo sapiens]"
16	S	EF2_HUMAN	43	2	"Elongation factor 2 OS=Homo sapiens OX=9606 GN=EEF2 PE=1 SV=4"
17	N	BAH12513.1	39	1	"unnamed protein product [Homo sapiens]"
18	N	AAF40160.1	36	1	"immunoglobulin heavy chain variable region 194-92 partial [Homo sapiens]"
19	S	TRFL_HUMAN	33	1	"Lactotransferrin OS=Homo sapiens OX=9606 GN=LTF PE=1 SV=6"
20	N	CAD38944.1	31	1	"hypothetical protein partial [Homo sapiens]"
21	S	KMT2A_HUMAN	26	1	"Histone-lysine N-methyltransferase 2A OS=Homo sapiens OX=9606 GN=KMT2A PE=1 SV=5"
22	S	MMP2_HUMAN	22	1	"72 kDa type IV collagenase OS=Homo sapiens OX=9606 GN=MMP2 PE=1 SV=2"
23	N	XP_011507815.3	21	1	"transport and Golgi organization protein 1 homolog isoform X3 [Homo sapiens]"
**F**	**DB**	**Accession**	**S**	**NM**	**S104–band 4–Description**
1	S	ALBU_HUMAN	422	21	"Serum albumin OS=Homo sapiens OX=9606 GN=ALB PE=1 SV=2"
1	N	CAA23753.1	142	13	"unnamed protein product [Homo sapiens]"
2	S	TRFE_HUMAN	113	4	"Serotransferrin OS=Homo sapiens OX=9606 GN=TF PE=1 SV=3"
3	N	SJM31191.1	63	5	"Sulfotransferase [Homo sapiens]"
4	N	AAC08449.1	56	2	"BIGH3 partial [Homo sapiens]"
5	N	CAD62334.1	50	1	"unnamed protein product partial [Homo sapiens]"
6	S	HEMO_HUMAN	41	1	"Hemopexin OS=Homo sapiens OX=9606 GN=HPX PE=1 SV=2"
7	N	CAD38944.1	40	1	"hypothetical protein partial [Homo sapiens]"
8	N	AKD42826.1	37	2	"anti-HIV-1 immunoglobulin light chain variable region partial [Homo sapiens]"
9	S	MCPH1_HUMAN	30	1	"Microcephalin OS=Homo sapiens OX=9606 GN=MCPH1 PE=1 SV=4"
10	S	MMP2_HUMAN	28	1	"72 kDa type IV collagenase OS=Homo sapiens OX=9606 GN=MMP2 PE=1 SV=2"
11	N	AAH38117.1	25	1	"LAPTM4B protein [Homo sapiens]"
12	S	CAD23_HUMAN	24	1	"Cadherin-23 OS=Homo sapiens OX=9606 GN=CDH23 PE=1 SV=2"
13	N	BAA91591.1	18	1	"unnamed protein product partial [Homo sapiens]"
14	S	LRP2_HUMAN	14	1	"Low-density lipoprotein receptor-related protein 2 OS=Homo sapiens OX=9606 GN=LRP2 PE=1 SV=3"
**F**	**DB**	**Accession**	**S**	**N**	**S104–band 5–Description**

				M	
1	S	PAI1_HUMAN	678	24	"Plasminogen activator inhibitor 1 OS=Homo sapiens OX=9606 GN=SERPINE1 PE=1 SV=1"
2	N	BAG62914.1	236	9	"unnamed protein product [Homo sapiens]"
3	S	ALBU_HUMAN	209	13	"Serum albumin OS=Homo sapiens OX=9606 GN=ALB PE=1 SV=2"
4	S	VIME_HUMAN	180	8	"Vimentin OS=Homo sapiens OX=9606 GN=VIM PE=1 SV=4"
5	S	SPRC_HUMAN	119	5	"SPARC OS=Homo sapiens OX=9606 GN=SPARC PE=1 SV=1"
6	S	TRFE_HUMAN	98	5	"Serotransferrin OS=Homo sapiens OX=9606 GN=TF PE=1 SV=3"
7	N	EAW68718.1	72	2	"hemopexin isoform CRA a [Homo sapiens]"
8	S	CO6A1_HUMAN	61	1	"Collagen alpha-1(VI) chain OS=Homo sapiens OX=9606 GN=COL6A1 PE=1 SV=3"
9	S	MMP1_HUMAN	60	1	"Interstitial collagenase OS=Homo sapiens OX=9606 GN=MMP1 PE=1 SV=3"
10	S	CLC11_HUMAN	47	1	"C-type lectin domain family 11 member A OS=Homo sapiens OX=9606 GN=CLEC11A PE=1 SV=1"
11	S	FSTL1_HUMAN	43	1	"Follistatin-related protein 1 OS=Homo sapiens OX=9606 GN=FSTL1 PE=1 SV=1"
12	S	PTX3_HUMAN	39	1	"Pentraxin-related protein PTX3 OS=Homo sapiens OX=9606 GN=PTX3 PE=1 SV=3"
13	N	SJM31191.1	39	2	"Sulfotransferase [Homo sapiens]"
14	S	ANT3_HUMAN	37	1	"Antithrombin-III OS=Homo sapiens OX=9606 GN=SERPINC1 PE=1 SV=1"
15	S	ISLR_HUMAN	34	1	"Immunoglobulin superfamily containing leucine-rich repeat protein OS=Homo sapiens OX=9606 GN=ISLR PE=2 SV=1"
16	N	CAD38944.1	26	1	"hypothetical protein partial [Homo sapiens]"
17	N	AAH38117.1	25	1	"LAPTM4B protein [Homo sapiens]"
18	S	TBA1B_HUMAN	25	1	"Tubulin alpha-1B chain OS=Homo sapiens OX=9606 GN=TUBA1B PE=1 SV=1"
19	S	ZC3H6_HUMAN	18	1	"Zinc finger CCCH domain-containing protein 6 OS=Homo sapiens OX=9606 GN=ZC3H6 PE=1 SV=2"
**F**	**DB**	**Accession**	**S**	**NM**	**S104–band 6–Description**
1	N	AAA51995.1	378	11	"alpha 1 (I) chain propeptide partial [Homo sapiens]"
2	S	ALBU_HUMAN	207	10	"Serum albumin OS=Homo sapiens OX=9606 GN=ALB PE=1 SV=2"
2	N	CAA23753.1	120	9	"unnamed protein product [Homo sapiens]"
3	N	BAG36698.1	177	5	"unnamed protein product [Homo sapiens]"
4	S	LDHA_HUMAN	164	4	"L-lactate dehydrogenase A chain OS=Homo sapiens OX=9606 GN=LDHA PE=1 SV=2"
5	S	CO1A2_HUMAN	137	6	"Collagen alpha-2(I) chain OS=Homo sapiens OX=9606 GN=COL1A2 PE=1 SV=7"

6	S	APOA1_HUMAN	117	2	"Apolipoprotein A-I OS=Homo sapiens OX=9606 GN=APOA1 PE=1 SV=1"
7	S	PSA2_HUMAN	103	2	"Proteasome subunit alpha type-2 OS=Homo sapiens OX=9606 GN=PSMA2 PE=1 SV=2"
8	S	TPM4_HUMAN	98	2	"Tropomyosin alpha-4 chain OS=Homo sapiens OX=9606 GN=TPM4 PE=1 SV=3"
9	S	1433B_HUMAN	95	1	"14-3-3 protein beta/alpha OS=Homo sapiens OX=9606 GN=YWHAB PE=1 SV=3"
10	S	PSA3_HUMAN	90	1	"Proteasome subunit alpha type-3 OS=Homo sapiens OX=9606 GN=PSMA3 PE=1 SV=2"
11	N	BAG60361.1	90	1	"unnamed protein product [Homo sapiens]"
12	S	1433Z_HUMAN	88	1	"14-3-3 protein zeta/delta OS=Homo sapiens OX=9606 GN=YWHAZ PE=1 SV=1"
13	S	TIMP1_HUMAN	86	1	"Metalloproteinase inhibitor 1 OS=Homo sapiens OX=9606 GN=TIMP1 PE=1 SV=1"
14	S	LDHB_HUMAN	76	2	"L-lactate dehydrogenase B chain OS=Homo sapiens OX=9606 GN=LDHB PE=1 SV=2"
15	S	ACTBL_HUMAN	71	1	"Beta-actin-like protein 2 OS=Homo sapiens OX=9606 GN=ACTBL2 PE=1 SV=2"
16	S	IBP7_HUMAN	68	2	"Insulin-like growth factor-binding protein 7 OS=Homo sapiens OX=9606 GN=IGFBP7 PE=1 SV=1"
17	S	TRFE_HUMAN	67	2	"Serotransferrin OS=Homo sapiens OX=9606 GN=TF PE=1 SV=3"
18	S	CLIC1_HUMAN	66	1	"Chloride intracellular channel protein 1 OS=Homo sapiens OX=9606 GN=CLIC1 PE=1 SV=4"
19	S	FBN1_HUMAN	63	1	"Fibrillin-1 OS=Homo sapiens OX=9606 GN=FBN1 PE=1 SV=3"
20	S	K1C10_HUMAN	59	1	"Keratin type I cytoskeletal 10 OS=Homo sapiens OX=9606 GN=KRT10 PE=1 SV=6"
21	S	PSA7_HUMAN	56	2	"Proteasome subunit alpha type-7 OS=Homo sapiens OX=9606 GN=PSMA7 PE=1 SV=1"
22	S	G3P_HUMAN	55	1	"Glyceraldehyde-3-phosphate dehydrogenase OS=Homo sapiens OX=9606 GN=GAPDH PE=1 SV=3"
23	N	SJM31191.1	54	4	"Sulfotransferase [Homo sapiens]"
24	S	PSA4_HUMAN	46	2	"Proteasome subunit alpha type-4 OS=Homo sapiens OX=9606 GN=PSMA4 PE=1 SV=1"
25	S	TPIS_HUMAN	42	1	"Triosephosphate isomerase OS=Homo sapiens OX=9606 GN=TPI1 PE=1 SV=3"
26	S	HEMO_HUMAN	38	1	"Hemopexin OS=Homo sapiens OX=9606 GN=HPX PE=1 SV=2"
27	S	PSA1_HUMAN	34	1	"Proteasome subunit alpha type-1 OS=Homo sapiens OX=9606 GN=PSMA1 PE=1 SV=1"
28	N	CAD38944.1	33	1	"hypothetical protein partial [Homo sapiens]"
29	S	TBA1B_HUMAN	32	1	"Tubulin alpha-1B chain OS=Homo sapiens OX=9606 GN=TUBA1B PE=1 SV=1"
30	S	RLA0L_HUMAN	32	1	"60S acidic ribosomal protein P0-like OS=Homo sapiens OX=9606 GN=RPLP0P6 PE=5 SV=1"

31	N	1IRU_E	32	1	"Chain E Crystal Structure Of The Mammalian 20s Proteasome At 2.75 A Resolution"
32	S	IGKC_HUMAN	29	1	"Immunoglobulin kappa constant OS=Homo sapiens OX=9606 GN=IGKC PE=1 SV=2"
[1] 33	N	AAH21119.1	26	1	"GAPVD1 protein [Homo sapiens]"

[1] LC-MS/MS: Liquid Chromatography Mass Spectrometry.
